# Altered dietary methionine differentially impacts glutathione and methionine metabolism in long-living growth hormone-deficient Ames dwarf and wild-type mice

**DOI:** 10.1186/2046-2395-3-10

**Published:** 2014-12-15

**Authors:** Holly M Brown-Borg, Sharlene Rakoczy, Joseph A Wonderlich, Vanessa Armstrong, Lalida Rojanathammanee

**Affiliations:** Department of Basic Sciences, University of North Dakota School of Medicine & Health Sciences, 501 N. Columbia Road, Grand Forks, ND 58203 USA; School of Sports Science, Institute of Science, Suranaree University of Technology, Muang District, Nakhon Ratchasima, 30000 Thailand

**Keywords:** Aging, Amino acids, Metabolomics, Ames mice, Longevity

## Abstract

**Background:**

Extending mammalian health span and life span has been achieved under a variety of dietary restriction protocols. Reducing the intake of a specific amino acid has also been shown to extend health and longevity. We recently reported that methionine (MET) restriction is not effective in life span extension in growth hormone (GH) signaling mutants. To better understand the apparent necessity of GH in the ‘sensing’ of altered dietary MET, the current study was designed to evaluate MET and glutathione (GSH) metabolism (as well as other pathways) in long-living GH-deficient Ames dwarf and wild-type mice following 8 weeks of restricted (0.16%), low (0.43%), or enriched (1.3%) dietary MET consumption. Metabolite expression was examined in liver tissue, while gene and protein expression were evaluated in liver, kidney, and muscle tissues.

**Results:**

Body weight was maintained in dwarf mice on the MET diets, while wild-type mice on higher levels of MET gained weight. Liver MET levels were similar in Ames mice, while several MET pathway enzymes were elevated regardless of dietary MET intake. Transsulfuration enzymes were also elevated in Ames mice but differences in cysteine levels were not different between genotypes. Dwarf mice maintained higher levels of GSH on MET restriction compared to wild-type mice, while genotype and diet effects were also detected in thioredoxin and glutaredoxin. MET restriction increased transmethylation in both genotypes as indicated by increased S-adenosylmethionine (SAM), betaine, and dimethylglycine. Diet did not impact levels of glycolytic components, but dwarf mice exhibited higher levels of key members of this pathway. Coenzyme A and measures of fatty acid oxidation were elevated in dwarf mice and unaffected by diet.

**Conclusions:**

This component analysis between Ames and wild-type mice suggests that the life span differences observed may result from the atypical MET metabolism and downstream effects on multiple systems. The overall lack of responsiveness to the different diets is well reflected across many metabolic pathways in dwarf mice indicating the importance of GH signaling in the ability to discriminate dietary amino acid levels.

**Electronic supplementary material:**

The online version of this article (doi:10.1186/2046-2395-3-10) contains supplementary material, which is available to authorized users.

## Background

Many successful efforts to extend mammalian health span and life span have involved a variety of dietary restriction (DR) protocols (decreasing calories consumed) applied to mice and rats. Altering the basic components in the diet has also been shown to affect physiological function and several parameters deemed important to health and longevity. In particular, it has been shown that reducing the amount of dietary methionine extends the life span of normal rats by more than 30% and extends life span in mice even when introduced midlife [1–4].

Long-living mutant mice have also benefitted from DR intervention in terms of longevity. Ames dwarf mice live more than 50% longer than their wild-type siblings when fed rodent chow ad libitum [5]. The dwarfism results from a point mutation in the Prop-1 gene that directs the differentiation of anterior pituitary. While these mice lack plasma growth hormone (GH), prolactin, and thyrotropin, the GH deficiency is primarily responsible for the life-extending effects [6]. Studies conducted by Bartke and coworkers demonstrated that a 30% DR increased life span by an additional 12% in these mice over Ames dwarf mice fed ad libitum [7]. These observations suggested that DR and GH deficiency exert different but overlapping effects that extend health and longevity.

The health benefits of DR interventions are shared in GH-deficient rodents and include improvements in insulin sensitivity, resistance to cellular/environmental stressors, reduced tumor incidence, delays in immune system aging, and the development of cataracts and osteoarthritis in many but not all studies [8–18]. Methionine restriction similarly appears to benefit animals by reducing age-related disorders and disease [1–3, 19–23].

The long-living Ames mouse exhibits atypical methionine (MET) metabolism. Multiple enzymatic components (mRNA, proteins, activities) of this pathway are upregulated in dwarf mice including methionine adenosyltransferase (Mat), glycine N-methyltransferase (Gnmt), S-adenosylhomocysteine hydrolase (Ahcy), cystathionine β-synthase (Cbs), and cystathionine γ-lyase (Cth) [24–26]. The flux of methionine through the transmethylation and transsulfuration pathways in liver and other tissues is also significantly higher in the Ames mice when compared to their wild-type controls [25]. Consequently, glutathione (GSH) is elevated in multiple tissues in young, middle-aged, and old mice [27, 28].

Recently, we conducted studies to examine the effects of different levels of dietary MET in the GH-deficient Ames dwarf and their GH-sufficient wild-type counterparts. We discovered that the Ames mice do not respond to changes in dietary MET in terms of life span, body weight, or food consumption when compared to wild-type mice [29]. To better understand the underlying metabolic responses to altered MET as it relates to GH status, we undertook studies to determine the components of the MET and GSH pathways in these animals following 8 weeks of dietary 0.16%, 0.43%, and 1.3% methionine. We found that in general, dwarf mice do not discriminate the differences in dietary MET with alterations in several metabolic pathways when compared to wild-type mice.

## Results

**Figure 1:**
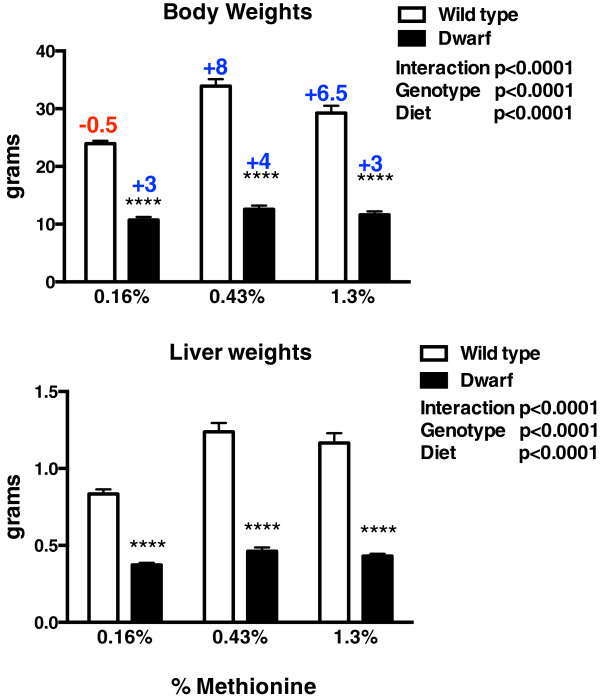
**Body and liver weights (grams) in Ames dwarf and wild**-**type mice following 8 weeks of 0.16%, 0.43%, or 1.3% dietary methionine consumption.** Values in bar graphs represent means ± SEM. Asterisks represent significant genotype differences within diet. ****p < 0.0001 dwarf versus wild-type mice. Colored numbers above each bar represent average gain or loss of weight from the beginning to the end of the 8-week period.

Dwarf mice maintained similar body weights regardless of MET content in the diet and remained significantly lower than wild-type mice (Figure 1). Across diets, dwarf mice gained on average 3.4 g over 8 weeks. Wild-type mice did not gain weight on the 0.16% MET diet (*X* = -0.53 g between days 1 and 56) but gained 8.3 and 6.6 g over 8 weeks on the 0.43% and 1.3% diets, respectively. Similarly, liver weights were maintained in dwarf mice across diets, while higher levels of MET resulted in heavier livers in wild-type mice corresponding with body weights (Figure 1).

The transcriptional, translational, and enzyme activity assays were conducted using tissues from each of the three diets; however, the metabolomic analysis was conducted using liver tissue from animals consuming the 0.16% and 1.3% MET diets only. Therefore, the data linked to these metabolites reflects values from the same diets. We evaluated the gene and protein expression of MET metabolic components as well as associated metabolites to understand the role of GH status. In previous studies, we have observed that mRNA levels of MET pathway components are highly reflective of activity levels in liver tissue. In addition, commercial antibodies were only available for some of the proteins of interest, so the corresponding protein data is limited.

**Figure 2:**
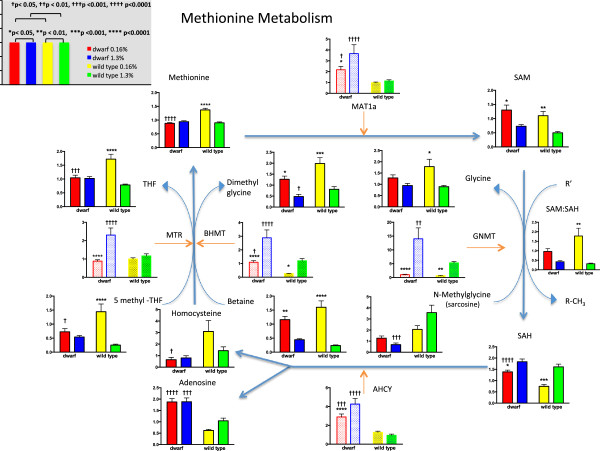
**Liver metabolite (solid bars; scaled intensity) and gene expression (shaded bars; relative expression) of methionine pathway components in Ames dwarf and wild**-**type mice following 8 weeks of 0.16% (red/yellow) or 1.3% (blue/green) dietary methionine consumption.** Asterisks represent significant differences between diets within a genotype. Crosses represent significant genotype differences within a diet. Values represent means ± SEM (*n* = 8/genotype/diet for metabolome data; *n* = 10–16/genotype/diet for gene expression data).

Changes in dietary MET intake are reflected in liver MET metabolism. Methionine levels in Ames mouse liver were similar regardless of dietary MET level (Figure 2). Wild-type mice on the MET-enriched diets had similar levels of MET to the dwarf, but MET was >50% higher in mice on the 0.16% MET level. N-acetylmethionine, a metabolic and nutritional equivalent of MET and reflective of MET levels, followed an identical pattern (Additional file 1). Methionine adenosyltransferase 1a (Mat1a) gene expression was 100–200% higher in GH-deficient dwarf mice across diets compared to diet-matched wild-type mice (Figure 2, Additional file 2). In addition, the 1.3% diet induced a 60% increase in Mat1a expression in dwarf liver compared to the 0.16% restricted MET level. The antibody available to examine Mat is not specific to the product of the Mat1a gene. However, in agreement with gene expression, Mat protein levels were higher in dwarf mice regardless of MET intake and increased in both genotypes on the highest level of MET (Additional file 3). Although the dwarf exhibits greater Mat enzyme levels, the level of S-adenosylmethionine (SAM) did not differ between the two genotypes (Figure 2). SAM levels were higher in both dwarf and wild-type mice on MET-restricted diets compared to MET-enriched diets (78% and 120%, dwarf and wild type, respectively). Gnmt metabolizes SAM to S-adenosylhomocysteine (SAH). Gnmt mRNA levels were markedly higher in dwarf mice on 0.43% and 1.3% MET compared to diet-matched wild-type mice (Additional file 2). Dwarf mice maintained significantly greater amounts (86%) of SAH in MET-restricted conditions compared to wild-type mice (Figure 2, Additional file 2). However, higher liver SAH levels were observed in both genotypes fed 1.3% MET compared to the 0.16% MET.

Ames mice maintained a nearly 1:1 (0.94) ratio of SAM to SAH on MET restriction, whereas in wild-type mice, this ratio was 1.49 (Figure 2). On enriched MET levels, the ratio was 0.40 in dwarf and 0.31 in wild-type mice. SAH is metabolized to homocysteine via Ahcy. The transcription of the Ahcy enzyme was greater in dwarf mice regardless of diet (Figure 2, Additional file 2). Liver homocysteine levels were 80% lower in dwarf mice on 0.16% MET compared to wild-type mice (Figure 2). Homocysteine was not affected by diet within genotype.

**Figure 3:**
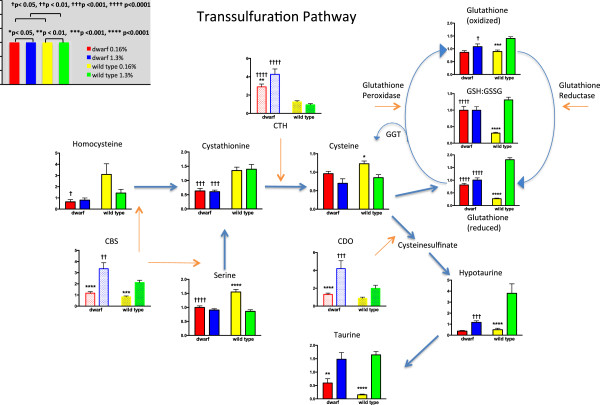
**Liver metabolite (solid bars; scaled intensity) and gene expression (shaded bars; relative expression) of transsulfuration, glutathione, and taurine pathway components in Ames dwarf and wild**-**type mice following 8 weeks of 0.16% (red/yellow) or 1.3% (blue/green) dietary methionine consumption.** Asterisks represent significant differences between diets within a genotype. Crosses represent significant genotype differences within a diet. Values represent means ± SEM (*n* = 8/genotype/diet for metabolome data; *n* = 10–16/genotype/diet for gene expression data).

Homocysteine is a juncture where the utilization of this factor can occur in either of two pathways (Figures 2 and 3). The recycling of homocysteine back to MET is supported by the metabolism of betaine to dimethylglycine, the folate process that converts 5-methyltetrahydrofolate (5-MeTHF) to tetrahydrofolate (THF) by methionine synthase (Mtr) and the cycling of THF back to 5-THF by 5,10-methylenetetrahydrofolate reductase (Mthfr). Mtr expression was similar between genotypes on the restricted and low (0.43%) MET diets but much higher in liver from dwarf animals consuming the MET-enriched food (Figure 2, Additional file 2). The levels of 5-MeTHF and THF were unchanged by dietary MET in Ames dwarf mice but were higher in wild-type mice on the 0.16% level of MET (Figure 2). The Mthfr enzyme was similarly elevated in dwarf mice at both the 0.16% and 1.3% MET diets (Additional file 2). Liver betaine (trimethylglycine) levels were significantly greater in both genotypes consuming 0.16% MET when compared to 1.3% MET but without genotype differences. Betaine homocysteine methyltransferase (Bhmt) transcript levels were 360% and 140% higher in dwarf mice on 0.16% and 1.3% MET compared to wild-type mice (Figure 2, Additional file 2). The product, dimethylglycine, mirrored betaine levels with higher amounts found in animals consuming 0.16% MET compared to MET-enriched diets. In addition, 50% more dimethylglycine was detected in wild-type mice compared to the dwarf with much lower gene expression of Bhmt.

If homocysteine is not recycled to MET, it flows irreversibly through the transsulfuration pathway and is acted upon by Cbs that combines serine and homocysteine to form cystathionine (Figure 3). The transcript and protein levels for liver Cbs were elevated in dwarf versus wild-type mice regardless of the MET content of the diet (Figure 3, Additional files 2 and 3). Transcript levels were higher in animals consuming the MET-enriched diet. Cystathionine, the resulting metabolite, was expressed at lower levels in dwarf mice for all diets when compared to wild-type mice possibly reflecting the very high transcript and protein levels of the enzyme that follows, Cth (Figure 3, Additional files 2 and 3). The gene expression of Cth in dwarf mice was 120% or higher in all diets, whereas protein levels were 96%, 210%, and 38% greater than wild-type mice on 0.16%, 0.43%, and 1.3% MET, respectively (Figure 3, Additional files 2 and 3). This enzyme is responsible for cysteine production. Interestingly, cysteine levels were not different between genotypes but higher in wild-type mice consuming the MET-restricted food compared to the MET-enriched food, reflecting higher Cth protein levels within wild-type mice across diets (Figure 3).

**Figure 4:**
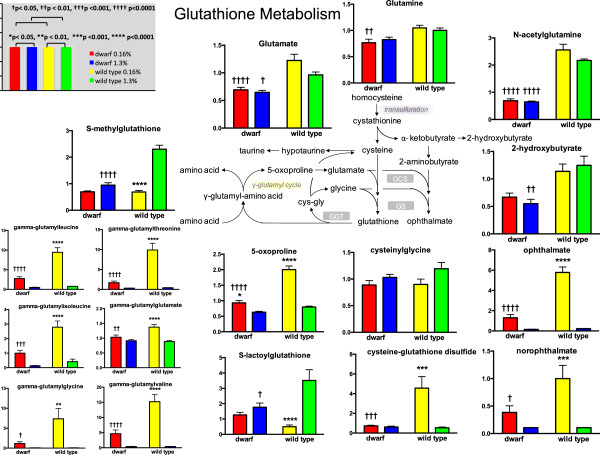
**Liver glutathione metabolites (scaled intensity) in Ames dwarf and wild**-**type mice following 8 weeks of 0.16% (red/yellow) or 1.3% (blue/green) dietary methionine consumption.** Asterisks represent significant differences between diets within a genotype. Crosses represent significant genotype differences within a diet. Values represent means ± SEM (*n* = 8/genotype/diet).

Cysteine generated via transsulfuration is utilized in the generation of proteins, hydrogen sulfide, taurine, and GSH. In GSH biosynthesis, cysteine is conjugated to glutamate by the enzyme γ-glutamyl cysteine ligase (GCL) to form γ-glutamyl cysteine. Dwarf mice had lower levels of glutamate compared to wild-type mice on both diets (Figure 4). GCL has two subunits that combine to create the active enzyme. The transcript levels of γ-glutamyl cysteine ligase catalytic subunit (Gclc) were twice as high in dwarf mice fed 0.16% and 1.3% MET when compared to wild-type mice (Additional file 2). This product in turn, is conjugated to glycine to form GSH. Glycine levels were not different between genotypes but greater in wild-type mice on the 0.16% MET diets compared to the 1.3% diet (Figure 2). Metabolomic analysis indicated that the resulting levels of reduced GSH were significantly lower in wild-type mice on MET restriction compared to the levels in wild-type mice fed 1.3% MET and to dwarf mice on either diet (Figure 3). Dwarf mice did not differ in reduced or oxidized GSH when fed different levels of MET. Similar to the reduced form, wild-type mouse liver from animals fed MET-enriched diets exhibited higher glutathione disulfide (GSSG) compared to those fed MET-restricted food. Our GSH/GSSG assay demonstrated that dwarf mice on 0.16% MET have higher GSH than wild-type mice, and GSH and GSSG were unaltered in these mice by different levels of MET confirming the metabolomic data (Additional file 4). The GSH:GSSG ratios were unaffected by diet in dwarf mice, whereas the wild-type mouse ratios were much lower on restricted MET compared to the enriched diets.

**Figure 5:**
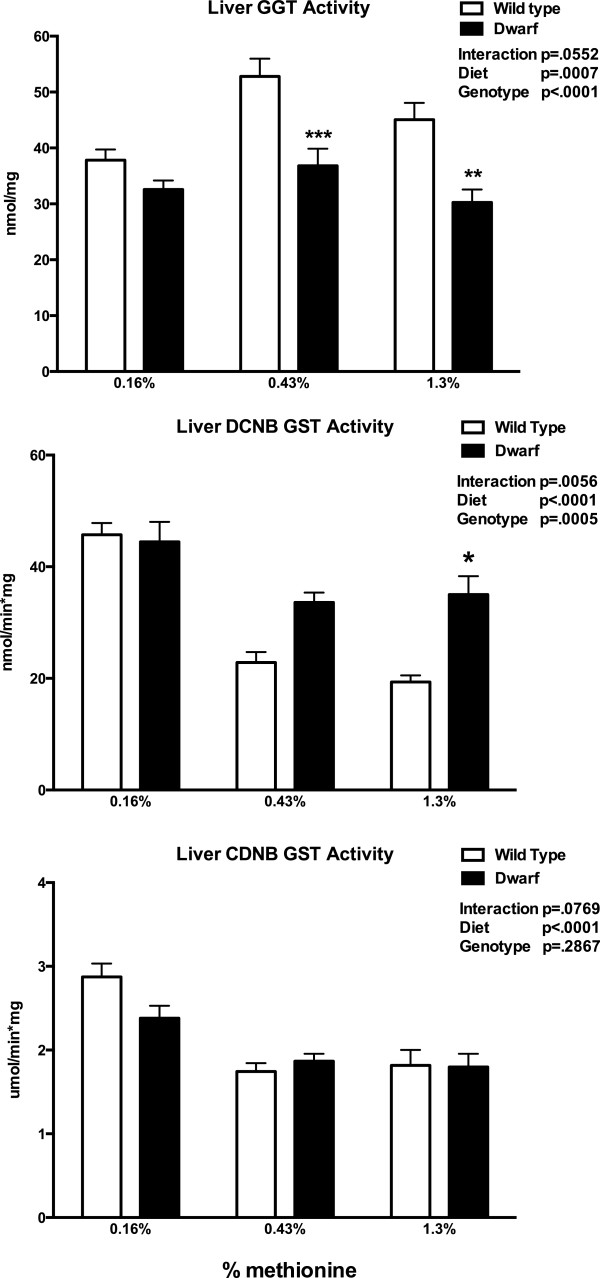
Liver glutathione S-transferase (GST; nmol/min*mg protein or μmole/min*mg protein) and γ-glutamyltranspeptidase (GGT; nmol/mg protein) activities in Ames dwarf and wild-type mice following consumption of 0.16%, 0.43%, or 1.3% methionine for 8 weeks. Values represent means ± SEM (*n* = 10–16/genotype/diet). *p < 0.05, **p < 0.01, ***p < 0.001, and ****p < 0.0001 dwarf versus wild-type mice.

An additional source of cysteine is derived from the degradation of GSH by γ-glutamyltranspeptidase (GGT). Liver GGT activity in this study revealed lower levels of activity in dwarf mice fed 0.43% and 1.3% MET when compared to wild-type mice, and diet appeared to also impact the level of GGT in this tissue (Figure 5). As a functional correlate of GSH utilization, we determined whether the activity of GSH S-transferase (GST) would be altered further by changes in MET intake. MET in the diets did not affect GST activity in dwarf mice, whereas wild-type mice lost activity with increasing concentrations of MET. Dwarf mice had higher DCNB GST activity than wild-type mice on 1.3% MET (Figure 5).

Cysteine is also utilized in the formation of another amino acid, taurine. Cysteine dioxygenase, the enzyme that metabolizes cysteine to cysteine sulfinate, was upregulated in dwarf compared to wild-type mice and increased in both genotypes with increasing MET content (Figure 3, Additional files 2 and 3). Hypotaurine levels were increased in wild-type mice on 1.3% MET compared to the dwarf (Figure 3), but dietary MET content did not affect hypotaurine in dwarf mice. Taurine levels were significantly increased in both genotypes on 1.3% MET compared to animals on the 0.16% MET, but genotype did not appear to play a role in the levels of this amino acid.

**Figure 6:**
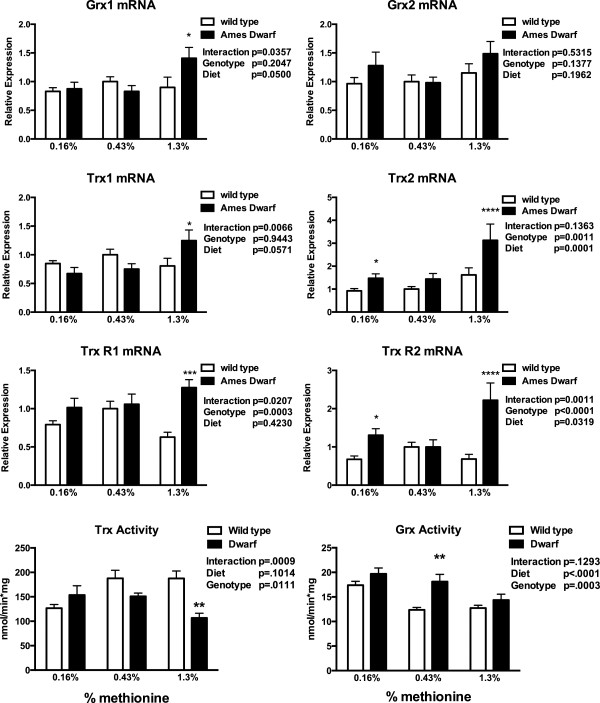
Gene expression (relative expression) and activity (nmol/min*mg protein) levels of liver glutaredoxin (Grx) and thioredoxin (Trx, TrxR) in Ames dwarf and wild-type mice following 8 weeks of dietary methionine consumption. Values represent means ± SEM (*n* = 10–16/genotype/diet). *p < 0.05, **p < 0.01, ***p < 0.001, and ****p < 0.0001 dwarf versus wild-type mice.

In past studies, the components of both oxidative and thiol pathways were shown to differ significantly between dwarf and wild-type mice; thus, the redoxin pathways were examined. In liver tissue, thioredoxin 1 (Trx1) and Trx2 in dwarf mice on the MET-enriched diets were elevated in comparison to diet-matched wild-type mice (Figure 6). In the Ames mouse, Trx2 levels were also higher at lower levels of MET. The reductases for the thioredoxins followed a very similar pattern with the dwarf mice exhibiting high levels on MET enrichment. When activity of Trx was examined, dwarf mice were unaffected by MET content in the diet but had lower levels of Trx activity compared to wild-type mice when consuming 1.3% MET (Figure 6). Dwarf mice consuming 1.3% MET exhibited greater glutaredoxin 1 (Grx1) mRNA levels, while the activity of Grx was greater across diets compared to wild-type mice.

## Discussion

When the consumption of the essential amino acid methionine is limited, the health span and life span extension of rodents is observed, similar to interventions reducing caloric intake. Ames dwarf mice live longer than their wild-type siblings when fed ad libitum rodent chow, and further extensions in life span are observed when this chow is reduced by 30% [5, 7]. However, in a recent report, we showed that dietary MET restriction did not impact life span in these GH-deficient mice as well as growth hormone receptor knockout mice suggesting that GH signaling is necessary to discriminate amino acid intake [29]. Indeed, the reductions of 50% and 80% MET as well as 150% supplementation did not alter life span in these mice nor was body weight influenced, contrary to several other reports in normal rodents.

In the current study, we fed Ames and wild-type mice different levels of MET and examined the corresponding metabolic pathways of MET, GSH, and redoxin to better understand the relationships between GH deficiency and amino acid metabolism. We focused on liver tissue as it is the primary organ involved in MET metabolism as well as GSH biosynthesis, detoxification, and stress resistance. We also evaluated kidney as this tissue is responsible for GSH degradation and detoxification of endogenous and exogenous substances and has an active transsulfuration system. Skeletal muscle served as a post-mitotic tissue with limited roles in each of these systems. The results of these two tissues are provided in Additional files 5, 6, 7, 8, 9, and 10 for tissue response comparison. The levels of MET were chosen based on previous literature showing that an 80% reduction extends life span (0.16%) in rodents. The 0.43% MET level represented a 50% reduction in typical rodent chow and a level that could be applied in human interventional studies. We also utilized supplementary MET (50% above typical levels of rodent diet) but incorporated a level well below that shown to be toxic (1.3%).

The body weights of dwarf mice were unaffected by dietary MET, similar to our previous report that examined lifetime body weight on these diets. The body and liver weight responses were reflected in the plasma insulin-like growth factor 1 (IGF1) levels that were shown to decrease in wild-type mice only as dietary MET levels decreased [29]. Other investigations have shown that body weights were either lower or unchanged by dietary MET restriction [1–3, 22].

In general, this study demonstrated that short-term diets containing restricted, low, or enriched MET produce vastly different liver metabolomic, mRNA, and protein profiles in wild-type mice. Dwarf mice, however, demonstrated fewer statistically significant differences between diets indicating that these mice do not respond in a similar manner to alterations in MET intake. This may reflect the differential MET metabolism in GH-deficient dwarf mice due to the inability to signal through the GH pathway. This lack of responsiveness to the diets on metabolite, gene, and protein levels is reflected in the lack of a difference in life span when dwarf mice are fed these diets throughout life (8 weeks to death) [29].

Dwarf mice did not alter liver MET or cysteine levels in response to different levels of MET intake nor were homocysteine levels changed in these long-living mice. Wild-type mice generated higher levels of MET, cysteine, and homocysteine on MET restriction compared to the MET-enriched diet (1.3%) and in comparison to dwarf mice (0.16%). This finding suggests the conservation of sulfur AA possibly through recycling as the genotype and diet differences in MET are reflected in the folate pathway (folate, THF, 5-methyl THF). In contrast, MET restriction decreased sulfur amino acids in the serum and liver of rats inducing hyperhomocysteinemia [30, 31]. Wild-type mouse levels of the sulfur AA did not differ from dwarf mice when fed a MET-enriched diet even though all of the measured MET metabolic enzymes differed significantly in dwarf mice fed high MET. The dwarf and its wild-type counterpart respond differently to MET restriction when compared to one another and to rats. In response to restricted MET, folate or one-carbon metabolism in wild-type mice was elevated resulting in increased levels of MET compared to higher MET diets and to MET restriction in dwarf mice. One report showed that post-transcriptional downregulation of Cbs occurs when animals are fed MET-deficient diets (no MET) indicating that a specific sensing system in mice determines when MET intake is low and signals the liver to activate a reduction in Cbs to conserve MET [32].

SAM is an important precursor for three key biochemical pathways: methylation, transsulfuration, and polyamine synthesis. Mat catalyzes the formation of SAM from MET and is upregulated in dwarf mice independent of diet agreeing with our earlier reports in these mice fed typical rodent chow [24, 25]. MET restriction elevated SAM in both genotypes regardless of the level of the Mat enzyme in the liver. Genotype differences in downstream methyltransferases resulted in elevated SAH levels in dwarf mice. SAH is metabolized to homocysteine and adenosine via Ahcy, an enzyme that remains elevated in Ames mice on both diets when compared to wild-type mice. The low homocysteine levels in dwarf mice were maintained, yet corresponding adenosine levels were elevated. These observations suggest that the actions of enhanced Ahcy increased the levels of both products in dwarf mice but that homocysteine was likely rapidly utilized via transsulfuration as evidenced by the elevated levels of downstream Cbs and Cth. We have previously shown that MET flux through transsulfuration in dwarf mouse liver occurs at three times the rate of wild-type mice [25]. Also, the activity of Ahcy is regulated in part by GH; thus, in the absence of this hormone, the metabolic pathway of homocysteine may be dysregulated [33]. The MET-conserving enzymes (MatI, Ahcy, Bhmt, Mtr, Mthfr) tend to be inhibited by their products and other metabolites, and rats fed ample MET exhibit reduced hepatic levels of these enzymes. In dwarf mice, the opposite effect is observed with high levels of each enzyme when consuming the MET-enriched diet. Wild-type mice appear to respond to the normal feedback inhibition of the MET-conserving enzymes. The MET-catabolizing enzymes, Gnmt, Cbs, and Cth, are normally activated by the cycle metabolites, and liver levels increase with additional MET intake. In our study, these enzymes were higher in dwarf mice consuming the MET-enriched diet, and Cth was also higher in the MET-restricted dwarf when compared to their wild-type counterparts. Ahcy has been categorized both as a MET-conserving and MET-catabolizing enzyme as it has intermediate properties. Under normal circumstances, the thermodynamics of this reaction ensure the conversion of excess homocysteine to SAH, unless the concentration of adenosine is limiting [34]. Adenosine is not limiting in the dwarf mouse in this study. Homocysteine levels are low in dwarf mice on both diets likely due to the upregulated gene expression and the activities of both the MET recycling and especially the transsulfuration pathways [24].

When MET is restricted, dwarf mice maintain lower levels of both homocysteine and serine, which combine to form cystathionine in reduced amounts, a reaction catalyzed by Cbs. Cbs levels were affected by diet and genotype, yet the resulting levels of cystathionine were unaffected by diet. Cystathionase, also known as γ-cysteine lyase (or CSE) expression was elevated in dwarf mice independent of diet and similar to that found in our previous reports suggesting further dysregulation of these pathways in Ames mice [24, 25]. Although liver cysteine levels did not appear to differ, the manner in which cysteine was utilized differed significantly by genotype. Cysteine is used for de novo GSH biosynthesis, hypotaurine, and taurine biosynthesis, and it is incorporated into a variety of proteins, as well as hydrogen sulfide. Our previous work showed that GH-deficient dwarf mice maintain higher levels of GSH compared to wild-type mice [35]. In the dwarf, GSH, GSSG, and the redox pair ratio were unaltered by diet. GSH levels in wild-type mice did not respond similarly but matched those reported in rats showing that hepatic GSH was low in animals fed restricted MET [1, 2]. Selected metabolite levels within the GSH pathway (including γ-glutamyl cycle) are supportive indicating few dietary effects in dwarf mice in contrast to wild-type mice (Figure 4). Additionally, the dwarf mice fed 0.16% MET maintained lower levels of many of these metabolites in comparison to the wild type further suggesting that the mutant mice do not discriminate changes in MET intake. GSH biosynthesis requires adequate amounts of cysteine, glutamate, and glycine to maintain appropriate levels, and each of these intersect at metabolic ‘centers’ that mirror sulfur and nitrogen metabolism, one-carbon metabolism, and overall energy levels in a tissue [36]. The availability of these precursors reflects the overall metabolic status of cells and tissues [37].

The γ-glutamyl cycle allows GSH to serve as a continuous source of cysteine, and thus appears to contribute to the stable GSH levels in dwarf mice independent of dietary MET intake [38]. In contrast, levels of the γ-glutamylamino acids, 5-hydroxyproline, and GSH are significantly impacted by MET intake in wild-type mice conserving cysteine during low MET intake and utilizing cysteine primarily for GSH and hypotaurine synthesis when MET levels are high. These findings are consistent with the increased recycling of GSH via the γ-glutamyl cycle. As dwarf mice maintain greater GSH under restricted MET along with the decreased demand for growth due to GH deficiency, it makes metabolic ‘sense’ that cysteine may be shifted toward defense and cellular protection mechanisms (GSH, GST…).

2-Hydroxybutyrate (2-OHB) is released as a byproduct when cystathionine is cleaved to the cysteine that is incorporated into GSH. Elevated 2-OHB and further metabolism to ophthalmate (a structural analog of GSH) and norophthalmate reveal that restricted dietary MET increased the abundance in wild-type mice, and enriched MET suppressed formation in both genotypes suggesting the conservation of cysteine for protein synthesis under conditions of MET restriction (Figure 4). During metabolic stress, cysteine is diverted from transmethylation (to form MET) into transsulfuration to form cystathionine. However, we know from MET flux studies that the rates of transmethylation and transsulfuration are two and three times greater, respectively, in dwarf versus wild-type mice [25]. The enhanced MET enzyme activity and mRNA expression help to explain the increased flux through the transmethylation and transsulfuration pathways. Thus, lower levels of 2-OHB, ophthalmate, and norophthalmate in dwarf mice consuming altered MET diets are not reflective of the enhanced liver Gclc mRNA observed in this study. This observation contributes to the hypothesis that deficient GH signaling undermines normal allosteric and regulatory mechanisms affecting multiple metabolic pathways.

The transmethylation and MET salvage (5-methylthioadenosine; MTA) pathways are also involved in the regulation of MET metabolism. Both SAM and MTA work to reduce pro-growth signals including decreasing the mitogenic effects of IGF1 [39]. MTA is part of the MET salvage pathway that involves polyamine synthesis. The levels of MTA tended to be lower in dwarf mice independent of diet (Additional file 1). Others have reported that mice with increased MTA have lower polyamine synthesis [40]. In turn, ornithine levels were decreased in MET-restricted dwarf mice compared to the wild type likely reflecting the known stimulatory effects of GH on ornithine decarboxylase activity and polyamine synthesis [41–43]. An increase in transmethylation in both genotypes consuming restricted MET is also supported by increased SAM, betaine, and dimethylglycine. In dwarf mice, other methyltransferases, such as Gnmt and Bhmt as well as DNA methyltransferases, are upregulated when fed normal chow [44, 45]. Thus, the metabolite data indicate that elevated methyltransferase enzyme function is maintained in dwarf mice and increased in wild-type mice consuming excess MET indicative of sensitivity to dietary MET in the presence of GH.

The first step toward cysteine utilization in the synthesis of hypotaurine, taurine, and H_2_S occurs via cysteine dioxygenase. In dwarf mice, increased cysteine dioxygenase (CDO), Cbs, and Cth suggest increased cysteine, although our metabolite analysis did not detect higher cysteine levels in these mice. Hepatic protein levels of CDO are significantly elevated in dwarf mice on normal rodent chow (Brown-Borg, unpublished data). CDO is expressed in high levels in rodent liver and kidney and is strongly regulated in response to cysteine availability such that in animals fed low protein; CDO is low (conserving cysteine) and is increased (up to 45-fold) in hepatic tissue of animals fed excess sulfur AA, to remove high cysteine and prevent cytotoxicity [46, 47]. The process of desulfuration via CDO dominates when cysteine is deficient (MET restriction), whereas oxidative catabolism dominates when cysteine is in excess (MET enrichment). When adequate sulfur AAs are provided to rats, two thirds of cysteine catabolism is oxidative (CDO) and one third undergoes desulfuration (Cbs, Cth). Both the oxidative and desulfurative mechanisms of cysteine metabolism were upregulated in Ames mice suggesting that more cysteine is being routed through these pathways.

In addition to the enhanced antioxidative enzyme defense observed in dwarf mice, protein glutathionylation likely plays a role in these mice in protecting protein thiols from oxidative-induced damage [48]. We have preliminary evidence indicating greater glutathionylation in Ames mice (Brown-Borg, unpublished data). Glutaredoxins remove glutathionylated modifications from proteins, are dependent on GSH pools, and function to maintain a reduced state of cysteines in cellular protein [49–51]. In this study, the elevated hepatic Grx, Trx, and thioredoxin reductase (TrxR) reflect similar relationships between dwarf and wild-type mice reported previously and indicate that altered MET intake does not perturbate this redox system markedly [52]. In addition, GH suppresses the activity of Grx and Trx [52]. These results support the hypothesis that deficient GH signaling heightens oxidant defense mechanisms.

When the liver metabolite analysis is expanded to other key pathways, one can observe a more global picture of genotype and diet effects (Additional files 11 and 12). Several components of glycolysis reveal significant genotype-specific responses. Glucose, glucose 6-phosphate, fructose 6-phosphate, the hexose diphosphates, and glycerol 3-phosphate were higher in dwarf compared to wild-type mice while other glycolytic metabolites (glycerate, PEP, 3-phosphoglycerate, lactate) were lower (Additional file 11). However, different levels of dietary MET did not impact levels of the measured glycolytic components in either genotype. These findings are in contrast to those reported in calorie restriction where the glycolytic pathway was downregulated, and acetyl CoA was unaltered when compared to ad libitum-fed animals [53]. Major components within the Krebs cycle appeared to be similarly unaffected by diet suggesting that MET intake does not impact their functioning, that metabolite levels are not indicative of function, or that the static picture we obtained did not capture potential alterations (Additional file 12).

Coenzyme A is an indispensible cofactor in all organisms functioning as an acyl carrier and carbonyl activating groups in key metabolic pathways including the Krebs cycle and fatty acid metabolism [54]. Many thiol-containing proteins are elevated in dwarf mice including GSH, metallothionein, GSH peroxidase, and selenoprotein R (Msrb), and now, we show that the thiol, coenzyme A, is also higher in dwarf mice regardless of diet (Additional file 12) [55]. These high levels are in concert with the enhanced fat utilization suggested by low respiratory quotients observed in dwarf mice [56, 57]. Long-term MET restriction increases energy expenditure limiting fat deposition despite increased food intake suggesting that these diets lead to increased metabolic flexibility [22]. Since dwarf mice already appear to rely on fat as a metabolic fuel (RQ < 1 and high VO_2_), an additional mechanism, such as restricted MET intake, may not further impact these systems. These conditions in dwarf mice likely reflect the increased energy demand for thermogenesis [57, 58]. Fatty acid metabolism was impacted by restricted MET in wild-type mice but not in dwarf mice suggesting that the reduced mobilization of fatty acids from lipid stores with low MET may be dependent on GH signaling. Glycerophospholipid metabolism was also driven by restricted dietary MET in a GH-dependent manner with several phospholipid metabolites elevated in MET restriction compared to MET enrichment in wild type but not in dwarf mice (data not shown). Supporting evidence indicating enhanced fatty acid oxidation is derived from studies showing that PGC1-α, PPAR-α, acyl-CoA oxidase (rate-limiting enzyme in β-oxidation), Cyp4a, and others are significantly elevated in dwarf versus wild-type mice [59–61].

Many aspects of mitochondrial function are altered in the long-living Ames mice. The OXPHOS enzymes are upregulated, liver mitochondrial H_2_O_2_ production is reduced, and both cytosolic and mitochondrial antioxidative enzymes are increased providing the basis for the lower oxidative damage levels to DNA and proteins [55, 62]. MET is sensitive to ROS-mediated oxidation, and the product MET sulfoxide is reduced back to MET by thioredoxin. The increased potential for repair of protein oxidative damage is suggested by higher Msrb protein levels and the increased Trx in dwarf mice [35]. The dwarf maintained lower levels of methionine sulfoxide levels possibly due to altered Trx and Grx (Additional file 1).

Several differences in metabolite levels that report on glucose metabolism were observed with diet in wild-type mice (Additional file 11). Glucose and the six-carbon glycolytic intermediates, fructose 6-phosphate, fructose 1,6-diphosphate, and myo-inositol (1,4 or 1,3)-diphosphate were lower with MET restriction compared to MET enrichment. Glucose polymers that arise from glycogen metabolism (maltopentaose, maltotetraose, maltotriose, and maltose) were significantly lower in wild-type mice consuming 0.16% MET compared to those fed 1.3% MET (data not shown). These metabolites are thought to be reflective of glycogen breakdown [63]. The pentose phosphate pathway metabolites (ribose-5-phosphate, ribulose-5-phosphate, xylulose-5-phosphate, ribulose) were also lower with the 0.16% diet (data not shown).

Although all of the liver metabolomic data is not presented, in total, the data indicates that dwarf mice are able to maintain their distinctive MET metabolism despite changes in MET levels as indicated by relatively few diet-specific alterations when compared to wild-type mice. The responsiveness of the wild-type mouse is reflected in changes in life span when fed altered MET and much like previous studies.

## Conclusions

In dwarf mice, the altered expression of key MET pathway enzymes results in a lack of dietary MET effects on homocysteine, serine, cysteine, cystathionine, GSH, hypotaurine, MET, THF, folate, and homoserine among others. This component analysis between dwarf and wild-type mice suggests that the life span differences observed in these mice may result from the atypical MET metabolism and downstream effects on multiple systems in dwarf mice. The increased MET flux, β-oxidation, and glycolytic flux are consistent with high CoA levels. One limitation of our study is that each parameter was measured once across genotypes on each diet representing a snapshot, whereas a more dynamic picture would include multiple measures over time. However, we believe that the overall lack of responsiveness to the different diets is well reflected across many metabolic pathways in the Ames mice. In addition, some of the differences observed may also reflect the differential composition of the specialized diets compared to data generated with animals fed standard rodent chow. Thus, the Ames mice exhibit a distinct metabolic signature during MET restriction and MET enrichment, differing from wild-type mice, yet the responsiveness to altered MET intake was minimal in comparison. We and others have shown that many of the MET pathway components as well as related pathways (antioxidative enzymes, GSH, stress resistance among others) are influenced by GH status. We believe that the lack of responsiveness of the Ames mice to altered MET intake, at least in part, results from their GH deficiency. Our results suggest that GH signaling impacts dietary amino acid responsiveness and ability to alter life span. Altered MET intake profoundly impacted liver metabolism in GH-sufficient wild-type mice only.

## Methods

### Animals

Ames dwarf mice were derived from a closed colony with a heterogeneous background (over 30 years) at the University of North Dakota (UND). Dwarf mice were generated by mating either homozygous (df/df) or heterozygous (df/+) dwarf males with carrier females (df/+). All mice were bred and maintained at the UND Center for Biomedical Research under controlled conditions of photoperiod (12:12 h light/dark cycle) and temperature (22 ± 1°C) and ad libitum access to food (birth to 8 weeks of age; Teklad #8640) and water. Male animals were started on the methionine diets at 8 weeks of age (in staggered cohorts). Animal procedures were reviewed and approved by the UND Institutional Animal Care and Use Committee following the Guide for the Care and Use of Laboratory Animals (NRC, 2011). A total of 40 wild-type and 38 dwarf mice were utilized for these studies. Body weights were determined immediately prior to tissue collection.

### Diets

Three levels of methionine (016%, 0.43%, 1.3% MET) were incorporated into amino acid-defined diets that were manufactured by Harlan Teklad based on the AIN-93 diet (TD.10230, TD.10231, TD.10232). In each of the diets, glutamic acid was adjusted to keep the diets isonitrogenous (suggested by manufacturer, Harlan Laboratories, Madison, WI). l-methionine was the only source of sulfur amino acids in these diets. A restricted level of 0.16% MET was used based on the literature demonstrating life span extension in rodents and to avoid issues of rectal prolapse and early mortality that have been previously reported [3]. The 0.43% MET diet represented about 50% of the MET found in many of the rodent chows, and a level of methionine that might be more achievable in human diets. An increase of 50% above normal chow levels represented an enriched MET diet (1.3%) yet supplementing this amino acid below the reported level of toxicity [64, 65]. Sulfathiazole supplementation was not provided in these diets.

### Methionine and glutathione metabolism

Liver tissue was collected from Ames dwarf and wild-type mice following 8 weeks on the diets (*n* = 10–16/genotype/diet), rapidly frozen, and stored at -80°C until analysis (food was not removed the night before collection). Kidney and hind limb skeletal muscle tissue were also collected from the same animals, but the data derived from these tissues is presented only in the additional files for comparison to liver. For enzyme assays, tissue samples were homogenized on ice with a Bullet Blender in buffer (20 mM MOPS, 300 mM sucrose, and 0.1 mM EDTA at pH 7.2; Next Advance, Averill Park, NY). Homogenates were centrifuged for 30 min at 13,000 g and the supernatant fractions used for analysis following the determination of protein concentrations. The supernatants were used to determine the enzyme activities of glutathione S-transferase, GGT, and glutaredoxin using the spectrophotometric method of Holmgren [66–69]. Thioredoxin activity was measured by its ability to reduce insulin disulfide in the presence of NADPH and thioredoxin reductase [69, 70]. For each assay, the absorbance was read at 340 nm, appropriate blanks were subtracted from total absorbance, and the activity was calculated. The cellular oxidation state was determined by measuring the ratio of the specific reduction/oxidation pair, GSH/GSSG using procedures described by Griffith and employed in our laboratory [26, 71].

Gene expression was evaluated in tissues using standard real-time RT-PCR techniques as previously reported [26, 50]. Total RNA was extracted and equal amounts of RNA for the gene of interest (all used annealing temperatures at 62°C) and the reference gene, β2 microglobulin (β2M-annealing at 60°C), were utilized to perform one-step real-time semi-quantitative PCR using a QuantiTect SYBR Green RT-PCR kit (Qiagen). The gene-specific forward and reverse primers utilized are listed in Table 1. Gene expression was determined as previously described using the comparative CT method. The amount of target DNA was normalized to the endogenous reference gene and compared relative to the control group (diet-matched wild-type mice).

**Table 1:** **Primer sets (5′-3′) used for real-time PCR analysis of gene expression**

Gene of interest	GenBank accession #	Forward primer	Reverse primer
Methionine adenosyltransferase 1a (Mat1a)	NM_133653	ctgaggcgctctggtgtc	tcctgcatgtactgaactgttacc
Glycine N-methyltransferase (Gnmt)	NM_010321	gctggacgtagcctgtgg	cacgctcatcacgctgaa
S-adenosylhomocysteine hydrolase (Ahcy)	NM_016661	ctgttggggttcacttcctg	acattcagcttgcccaggt
Cystathionine β-synthase (Cbs)	NM_144855	cgcacaggaaggactgcta	agccttcacagccacagc
Cystathionase (Cth)	NM_145953	gagtctggctgagcttcca	cgagggtagctctgtccttc
Betaine homocysteine S-methyltransferase (Bhmt)	NM_016668	acgtggacttcctcattgcagagt	tgctacgggcttaccagatgcttt
5-Methyltetrahydrofolate-homocysteine methyltransferase (5-MeTHF-hmt)	XM_138431	gcagatgtggccagaaaag	gccacaaacctcttgactcc
5,10-Methylenetetrahydrofolate reductase (Mthfr)	NM_010840	agcttgaagccacctggactgtat	agactagcgttgctgggtttcaga
Glutamylcysteine ligase catalytic subunit (Gclc)	NM_010295	ggaggcgatgttcttgagac	cagagggtcggatggttg
Glutamylcysteine ligase modifier subunit (Gclm)	NM_008129	gactcacaatgacccgaaaga	gatgctttcttgaagagcttcct
Thioredoxin 1 (Trx1)	X77585	cgtggtggacttctctgctacgtggtg	ggtcggcatgcatttgacttcacagtc
Thioredoxin 2 (Trx2)	U85089	gctagagaagatggtcgccaagcagca	tcctcgtccttgatccccacaaacttg
Thioredoxin reductase 1 (TrxR1)	AB027565	ggccaacaaaatcggtgaacacatggaag	cgccagcaacactgtgttaaattcgccct
Thioredoxin reductase 2 (TrxR2)	AB027566	gtcccctcccacatcaaaaaactcccaac	ggcccacaggacagtgtcaaaggtgc
Glutaredoxin 1 (Grx1)	AB013137	tgcagaaagacccaagaaatcctcagtca	tggagattagatcactgcatccgcctatg
Glutaredoxin 2 (Grx2)	NM_023505	catcctgctcttactgttccatggccaa	tcatcttgtgaagcgcatcttgaaactgg
β2-microglobulin (B2M)	NM_00975	atgggaagccgaacatactg	cagtctcagtgggggtgaat

Immunoblotting was utilized to evaluate protein levels of various components of the glutathione and methionine pathways. Standard techniques previously developed and published were employed [44, 52]. Antibodies to mouse Gclm, Ahcy, Cbs, Mat, Trx1, Trx2, TrxR1, and TrxR2 were obtained from Proteintech (Chicago, IL). Bhmt, Cth, and Gnmt antibodies were obtained from Santa Cruz Biotechnology (Santa Cruz, CA), and Grx was purchased from R & D Systems (Minneapolis, MN). The CDO antibody was supplied by Abcam (Cambridge, MA). Chemiluminescence (Bio-Rad; Hercules, CA) and densitometry (Omega-Lum, Aplegen) were used for the detection and analysis of protein levels [28]. The Ponceau-S staining of membranes was used to evaluate equal loading of protein.

### Metabolomics

To gain more specific information regarding the response of the liver tissue to the effects of dietary methionine, we examined a broad spectrum of molecules using a non-targeted metabolomic approach. Liver tissues from animals consuming the 0.16% and 1.3% MET diets (*n* = 8/genotype/diet) were evaluated by Metabolon, Inc. (Research Triangle Park, NC). The tissues were processed using published procedures for protein precipitation and aliquoted for evaluation using three different mass spectrometry platforms [72, 73]. The liquid chromatography/mass spectrometry (LC/MS) was performed using a Waters Acquity UPLC system and a Thermo-Finnigan LTQ mass spectrometer, including an electrospray ionization (ESI) source and linear ion trap (LIT) mass analyzer. Aliquots of each vacuum-dried sample were reconstituted, one each in acidic or basic LC-compatible solvents containing eight or more injection standards at fixed concentrations (to both ensure injection and chromatographic consistency). Extracts were loaded onto columns (Waters UPLC BEH C18-2.1 × 100 mm, 1.7 μm) and gradient eluted with water and 95% methanol containing 0.1% formic acid (acidic extracts) or 6.5 mM ammonium bicarbonate (basic extracts). Samples for gas chromatography/MS (GC/MS) analysis were dried under vacuum desiccation overnight prior to being derivatized under nitrogen using bistrimethyl-silyl-trifluoroacetamide (BSTFA). Derivatized samples were separated on a 5% diphenyl/95% dimethyl polysiloxane fused silica column (20 m × 0.18 mm ID; 0.18 μm film thickness) with helium as carrier gas and a temperature ramp from 60°C to 340°C in a 17.5-min period and then analyzed on a Thermo-Finnigan Trace DSQ fast-scanning single-quadrupole mass spectrometer using electron impact (EI) ionization. The mass accuracy and calibration of the instrument were checked daily. Quality control measures included using columns and reagents from a single lot to complete all related experiments, the randomization of experimental samples and controls throughout each day’s run, the use of pooled experimental samples serving as technical replicates (for precision calculations) throughout the analyses, extracted water samples serving as process blanks, and a cocktail of standards spiked into every sample to monitor instrument performance.

The metabolites were identified by comparing the experimental samples to a reference library of purified chemical standards using chromatographic properties and mass spectra matching to the specific compound or an isobaric entity using proprietary visualization and interpretation software [74]. Library matches for each compound were verified for each sample. The liver metabolome consisted of 348 named biochemicals. In this analysis, the median relative standard deviation for the internal standards (instrument variability) was 4% and for the endogenous biochemicals (total process variability) was 12%.

### Statistics

For the gene expression, immunoblotting, activity assays, and metabolomic data comparisons, a two-way analysis of variance and, when appropriate, Bonferroni post hoc testing as well as Tukey’s were used to determine significant differences among means (Prism GraphPad, San Diego, CA). Data are reported as means ± SEM.

## Electronic supplementary material

Additional file 1: Figure S1: Liver metabolites (scaled intensity) of supporting components in Ames dwarf and wild-type mice following 8 weeks of 0.16% (red/yellow) or 1.3% (blue/green) dietary methionine consumption. Asterisks represent significant differences between diets within a genotype. Crosses represent significant genotype differences within a diet. Values represent means ± SEM (*n* = 8/genotype/diet for metabolome data). (PDF 208 KB)

Additional file 2: Figure S2: Liver gene expression (relative expression) of methionine, transsulfuration, and glutathione pathway components in Ames dwarf and wild-type mice following 8 weeks of 0.16%, 0.43%, or 1.3% dietary methionine consumption. Values represent means ± SEM (*n* = 10–16/genotype/diet). *p < 0.05, **p < 0.01, ***p < 0.001, and ****p < 0.0001 dwarf versus wild-type mice. (PDF 46 KB)

Additional file 3: Figure S3: Liver protein levels (relative optical density units) of Mat, Cbs, Cth, Gclm, and CDO in Ames dwarf and wild-type mice following 8 weeks of 0.16%, 0.43%, or 1.3% dietary methionine consumption. Values represent means ± SEM (*n* = 11–12/genotype/diet). *p < 0.05, **p < 0.01, ***p < 0.001, and ****p < 0.0001 dwarf versus wild-type mice. (PDF 34 KB)

Additional file 4: Table S4: Liver, kidney, and skeletal muscle glutathione (GSH) and glutathione disulfide (GSSG) in Ames dwarf and wild-type mice following 8 weeks of dietary methionine consumption (0.16%, 0.43%, 1.3%). Values represent means ± SEM (*n* = 10–16/genotype/diet). *p < 0.05, **p < 0.01, and ****p < 0.0001 dwarf versus wild-type mice. (PDF 61 KB)

Additional file 5: **Supporting information.** Supporting text for Additional files 6, 7, 8, 9, and 10 describing the kidney and muscle tissue responses to altered MET diet consumption. (PDF 50 KB)

Additional file 6: Figure S6: Kidney gene expression (relative expression) of methionine, transsulfuration, and glutathione pathway components in Ames dwarf and wild-type mice following 8 weeks of 0.16%, 0.43%, or 1.3% dietary methionine consumption. Values represent means ± SEM (*n* = 10–16/genotype/diet). *p < 0.05, **p < 0.01, ***p < 0.001, and ****p < 0.0001 dwarf versus wild-type mice. (PDF 39 KB)

Additional file 7: Figure S7: Kidney glutathione S-transferase (GST; nmol/min*mg protein) and γ-glutamyltranspeptidase (GGT; nmol/min*mg protein) activities in Ames dwarf and wild-type mice following consumption of 0.16%, 0.43%, or 1.3% methionine for 8 weeks. Values represent means ± SEM (*n* = 11–16/genotype/diet). **p < 0.01 and ****p < 0.0001 dwarf versus wild-type mice. (PDF 24 KB)

Additional file 8: Figure S8: Gene expression (relative expression) and activity (nmol/min*mg protein) levels of kidney glutaredoxin (Grx) and thioredoxin (Trx, TrxR) in Ames dwarf and wild-type mice following 8 weeks of dietary methionine consumption. Values represent means ± SEM (*n* = 10–16/genotype/diet). *p < 0.05, **p < 0.01, ***p < 0.001, and ****p < 0.0001 dwarf versus wild-type mice. (PDF 67 KB)

Additional file 9: Figure S9: Skeletal muscle gene expression (relative expression) of methionine pathway components in Ames dwarf and wild-type mice following 8 weeks of 0.16%, 0.43%, or 1.3% dietary methionine consumption. Values represent means ± SEM (*n* = 10–16/genotype/diet). *p < 0.05, **p < 0.01, ***p < 0.001, and ****p < 0.0001 dwarf versus wild-type mice. (PDF 38 KB)

Additional file 10: Figure S10: Gene expression (relative expression) levels of skeletal muscle glutaredoxin (Grx) and thioredoxin (Trx, TrxR) in Ames dwarf and wild-type mice following 8 weeks of dietary methionine consumption. Values represent means ± SEM (*n* = 10–16/genotype/diet). *p < 0.05, **p < 0.01, and ****p < 0.0001 dwarf versus wild-type mice. (PDF 30 KB)

Additional file 11: Figure S11: Liver glycolysis and pentose phosphate metabolites (scaled intensity) in Ames dwarf and wild-type mice following 8 weeks of 0.16% (red/yellow) or 1.3% (blue/green) dietary methionine consumption. Asterisks represent significant differences between diets within a genotype. Crosses represent significant genotype differences within a diet. Values represent means ± SEM (*n* = 8/genotype/diet). (PDF 208 KB)

Additional file 12: Figure S12: Liver Krebs cycle metabolites (scaled intensity) in Ames dwarf and wild-type mice following 8 weeks of 0.16% (red/yellow) or 1.3% (blue/green) dietary methionine consumption. Asterisks represent significant differences between diets within a genotype. Crosses represent significant genotype differences within a diet. Values represent means ± SEM (*n* = 8/genotype/diet). (PDF 214 KB)

## Abbreviations

MET: methionine
GH: growth hormone
GSH: glutathione
DR: dietary restriction
Mat: methionine adenosyltransferase
Gnmt: glycine N-methyltransferase
Ahcy: adenosylhomocysteine hydrolase
Cbs: cystathionine β-synthase
Cth: cystathionase
SAM: S-adenosylmethionine
SAH: S-adenosylhomocysteine
5-MeTHF: 5-methyltetrahydrofolate
THF: tetrahydrofolate
Mtr: methionine synthase
Mthfr: 5,10-methylenetetrahydrofolate reductase
Bhmt: betaine homocysteine methyltransferase
GCL: γ-glutamyl cysteine ligase
Gclc: γ-glutamyl cysteine ligase catalytic subunit
Gclm: γ-glutamyl cysteine ligase modifier subunit
GSSG: glutathione disulfide
GGT: γ-glutamyltranspeptidase
GST: glutathione S-transferase
Trx: thioredoxin
TrxR: thioredoxin reductase
Grx: glutaredoxin
CDO: cysteine dioxygenase
IGF1: insulin-like growth factor 1
2-OHB: 2-hydroxybutyrate
MTA: 5-methylthioadenosine
PEP: phosphoenolpyruvate
CoA: coenzyme A
RQ: respiratory quotient.
