# Supplementary figures and images for: Altered dietary methionine differentially impacts glutathione and methionine metabolism in long-living growth hormone-deficient Ames dwarf and wild-type mice
Source: Longev Healthspan. 2014 Dec 15;3:10. doi: 10.1186/2046-2395-3-10 (PMC4290132; doi:10.1186/2046-2395-3-10)

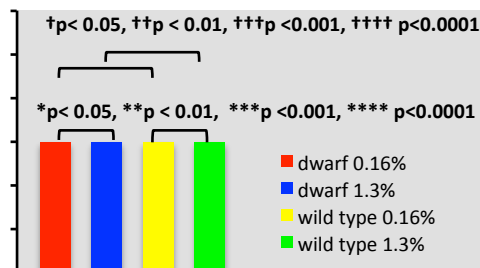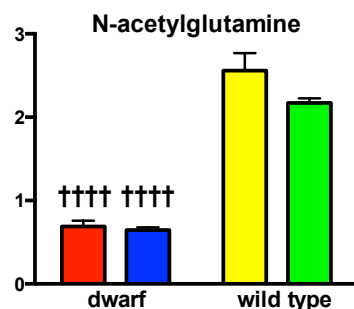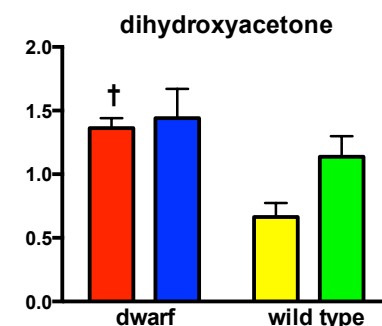

## Methionine salvage & polyamine synthesis

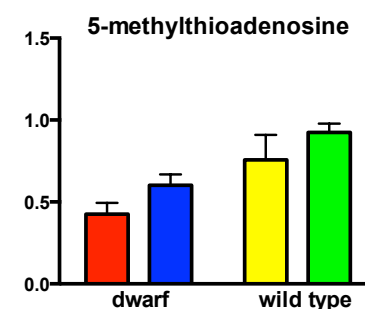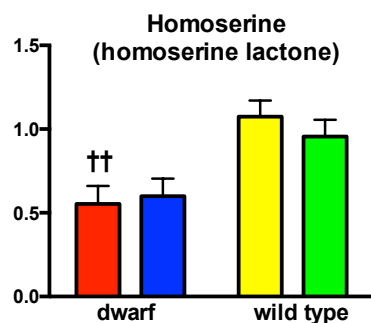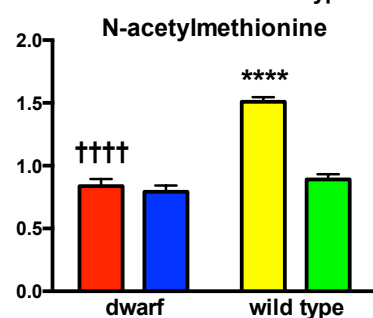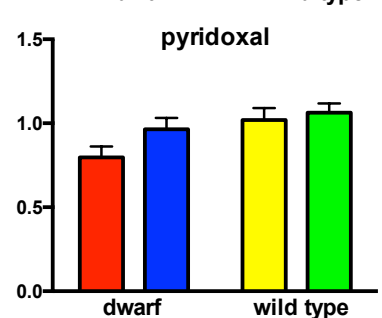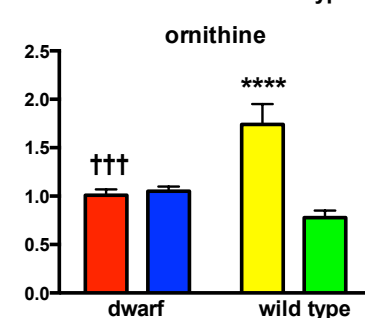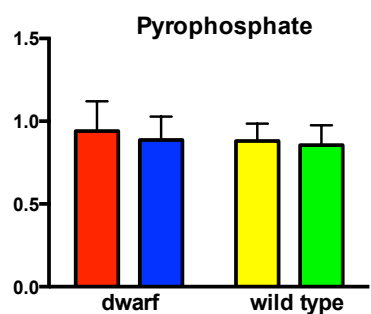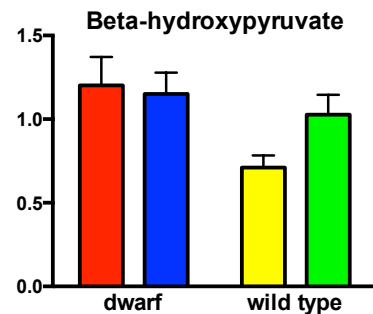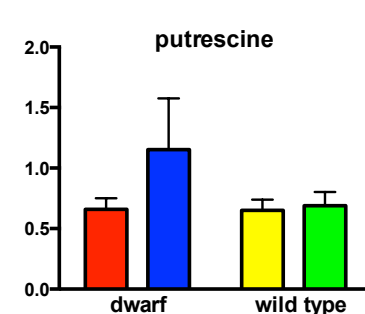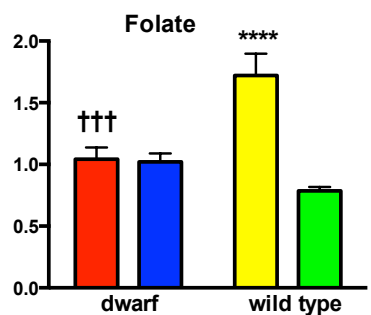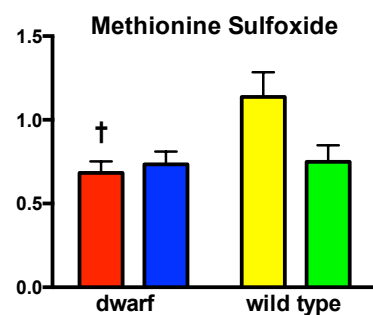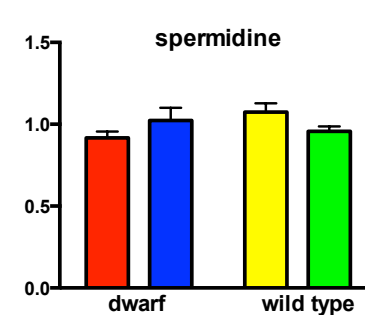

Supplement: Supplementary file 1 — Additional file 1: Figure S1: Liver metabolites (scaled intensity) of supporting components in Ames dwarf and wild-type mice following 8 weeks of 0.16% (red/yellow) or 1.3% (blue/green) dietary methionine consumption. Asterisks represent significant differences between diets within a genotype. Crosses represent significant genotype differences within a diet. Values represent means ± SEM (n = 8/genotype/diet for metabolome data). (PDF 208 KB) [file 13685_2014_31_MOESM1_ESM.pdf]

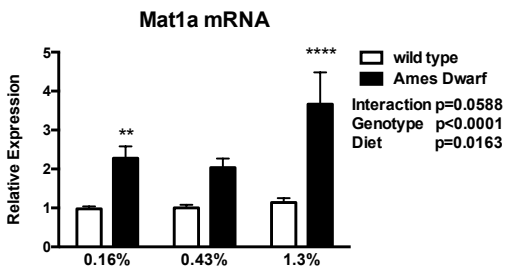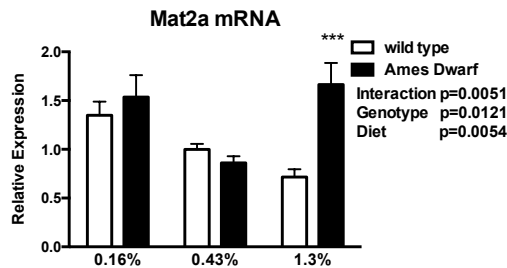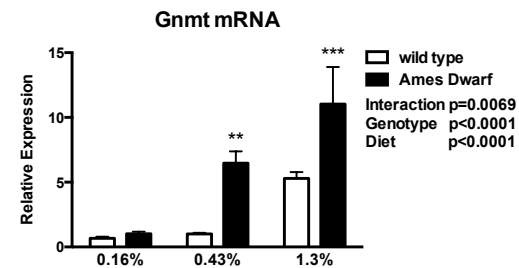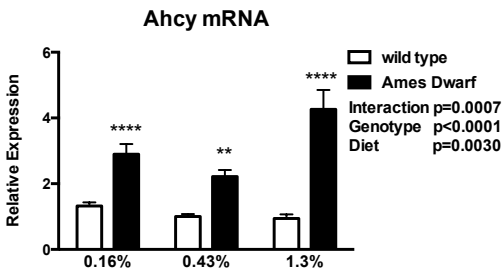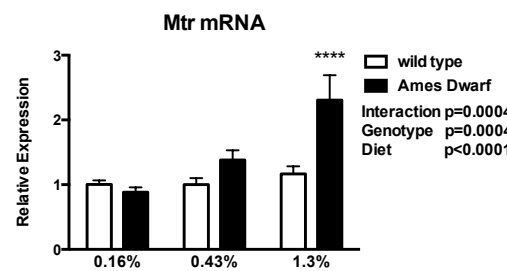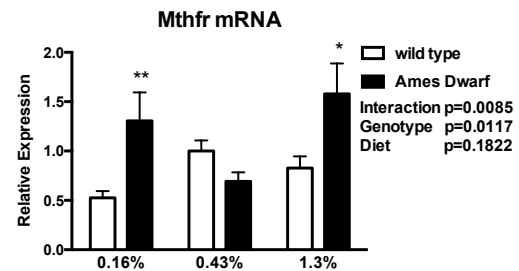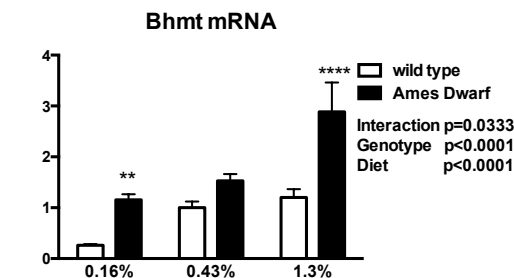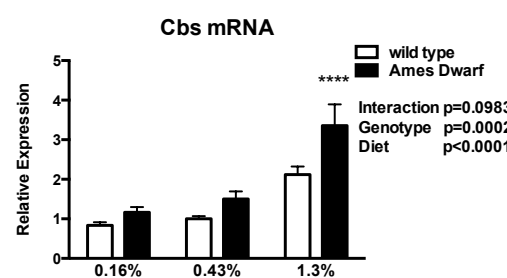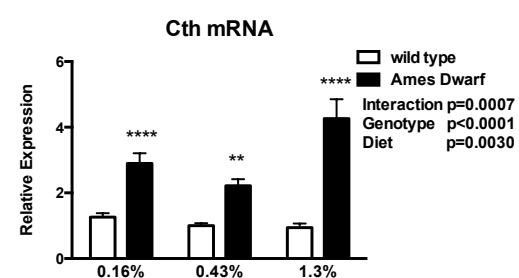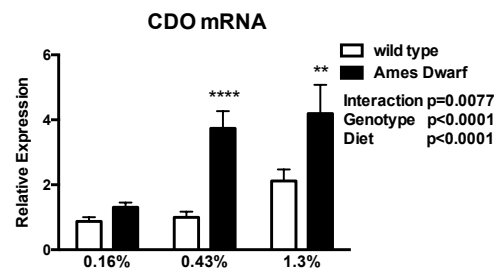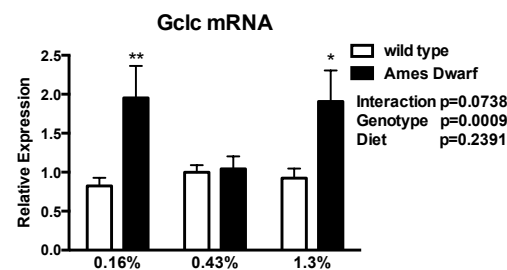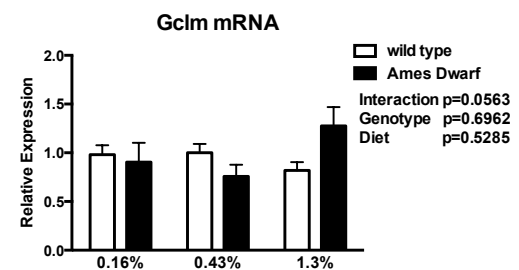

% methionine

% methionine

% methionine

Supplement: Supplementary file 2 — Additional file 2: Figure S2: Liver gene expression (relative expression) of methionine, transsulfuration, and glutathione pathway components in Ames dwarf and wild-type mice following 8 weeks of 0.16%, 0.43%, or 1.3% dietary methionine consumption. Values represent means ± SEM (n = 10–16/genotype/diet). *p < 0.05, **p < 0.01, ***p < 0.001, and ****p < 0.0001 dwarf versus wild-type mice. (PDF 46 KB) [file 13685_2014_31_MOESM2_ESM.pdf]

Liver MATI-II-III

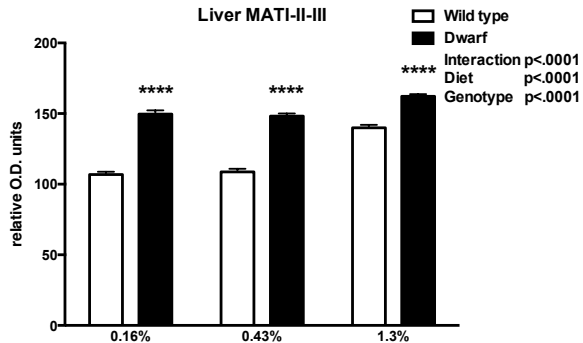

Liver Cbs

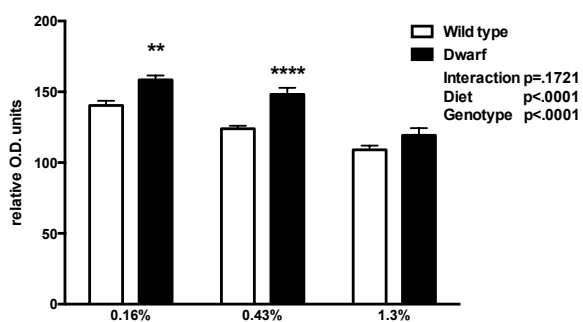

Liver Cth

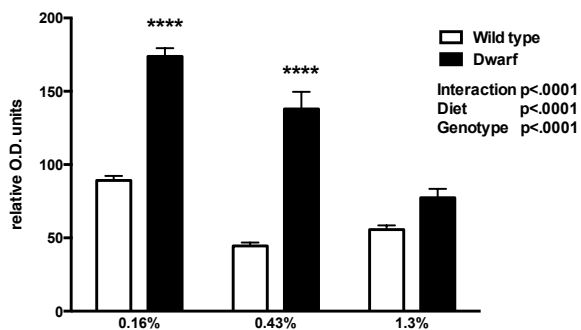

Liver GCLM

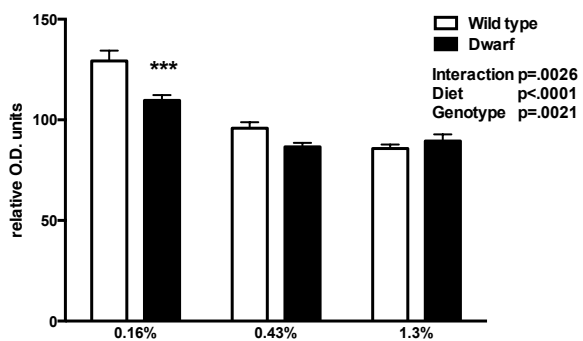

Liver CDO

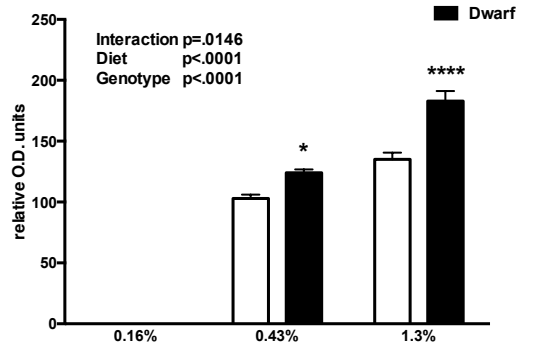

% methionine

% methionine

Supplement: Supplementary file 3 — Additional file 3: Figure S3: Liver protein levels (relative optical density units) of Mat, Cbs, Cth, Gclm, and CDO in Ames dwarf and wild-type mice following 8 weeks of 0.16%, 0.43%, or 1.3% dietary methionine consumption. Values represent means ± SEM (n = 11–12/genotype/diet). *p < 0.05, **p < 0.01, ***p < 0.001, and ****p < 0.0001 dwarf versus wild-type mice. (PDF 34 KB) [file 13685_2014_31_MOESM3_ESM.pdf]

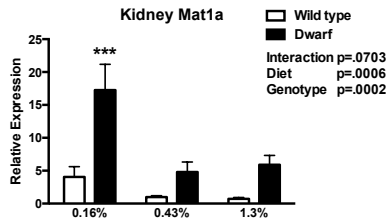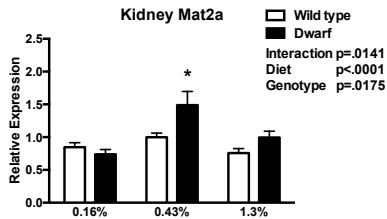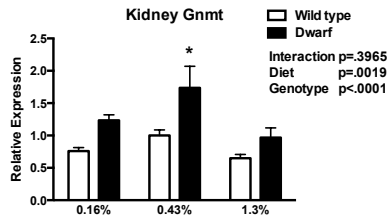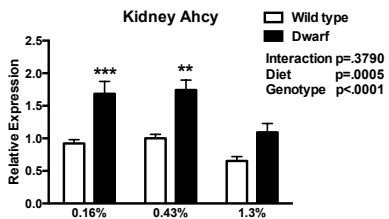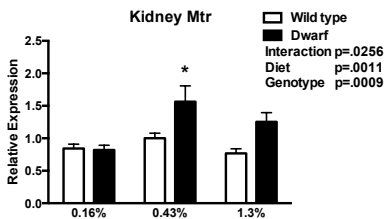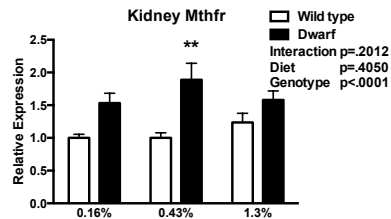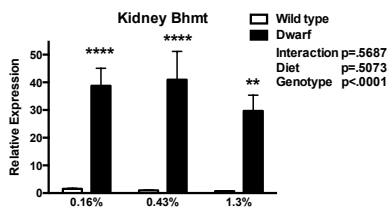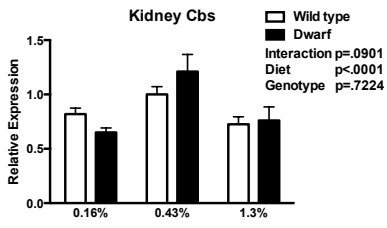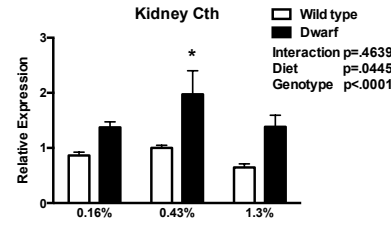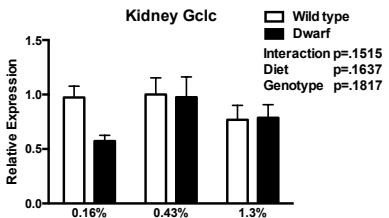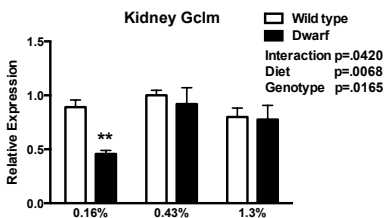

% methionine

% methionine

Supplement: Supplementary file 6 — Additional file 6: Figure S6: Kidney gene expression (relative expression) of methionine, transsulfuration, and glutathione pathway components in Ames dwarf and wild-type mice following 8 weeks of 0.16%, 0.43%, or 1.3% dietary methionine consumption. Values represent means ± SEM (n = 10–16/genotype/diet). *p < 0.05, **p < 0.01, ***p < 0.001, and ****p < 0.0001 dwarf versus wild-type mice. (PDF 39 KB) [file 13685_2014_31_MOESM6_ESM.pdf]

### Kidney GGT Activity

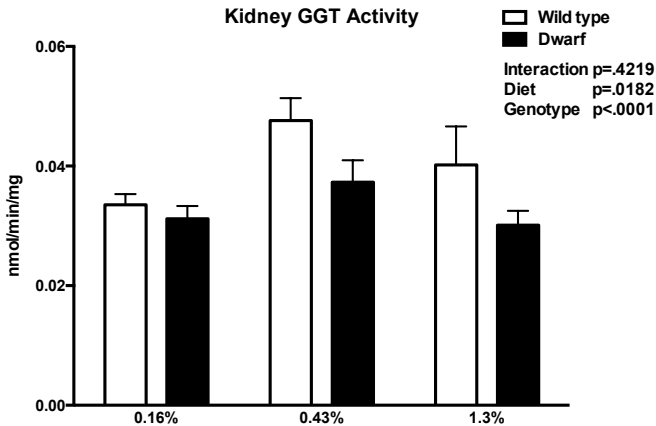

### Kidney GST CDBN Activity

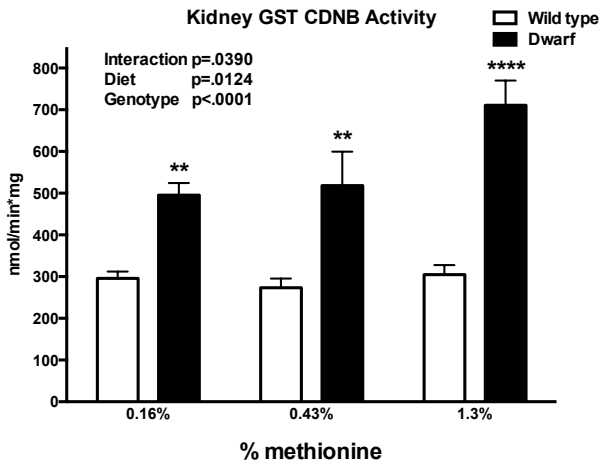

Supplement: Supplementary file 7 — Additional file 7: Figure S7: Kidney glutathione S-transferase (GST; nmol/min*mg protein) and γ-glutamyltranspeptidase (GGT; nmol/min*mg protein) activities in Ames dwarf and wild-type mice following consumption of 0.16%, 0.43%, or 1.3% methionine for 8 weeks. Values represent means ± SEM (n = 11–16/genotype/diet). **p < 0.01 and ****p < 0.0001 dwarf versus wild-type mice. (PDF 24 KB) [file 13685_2014_31_MOESM7_ESM.pdf]

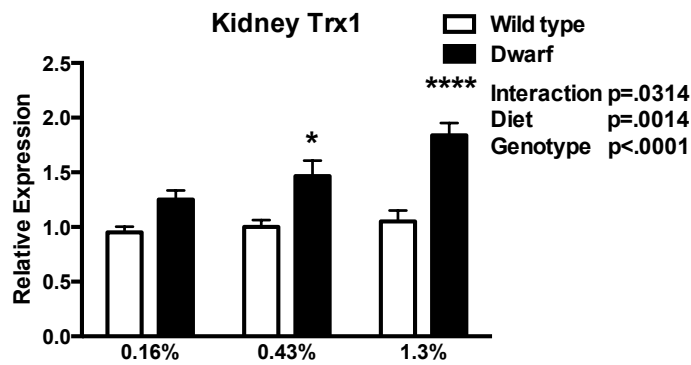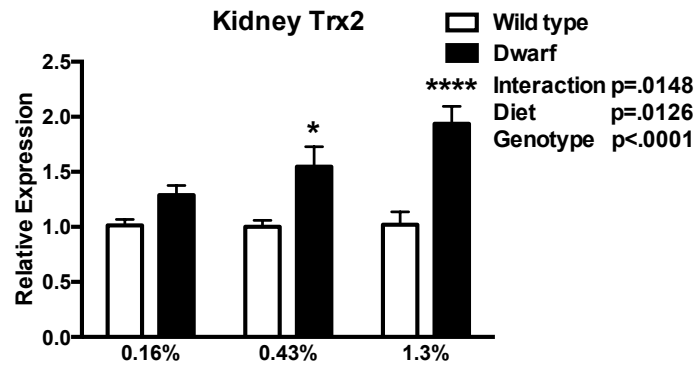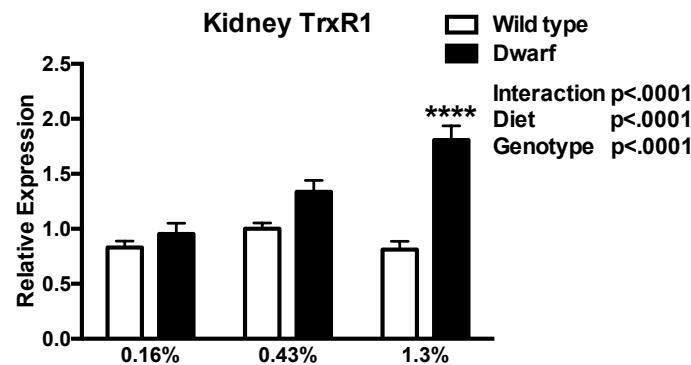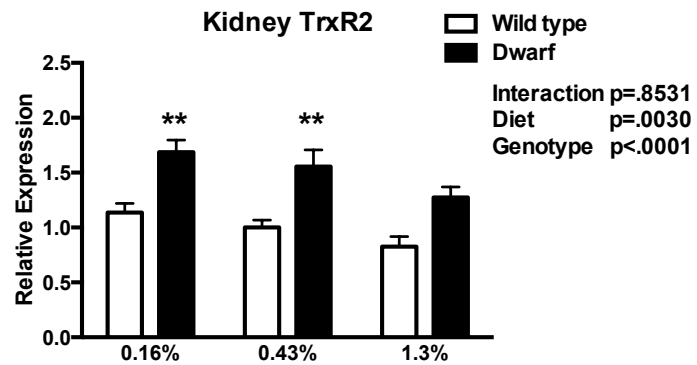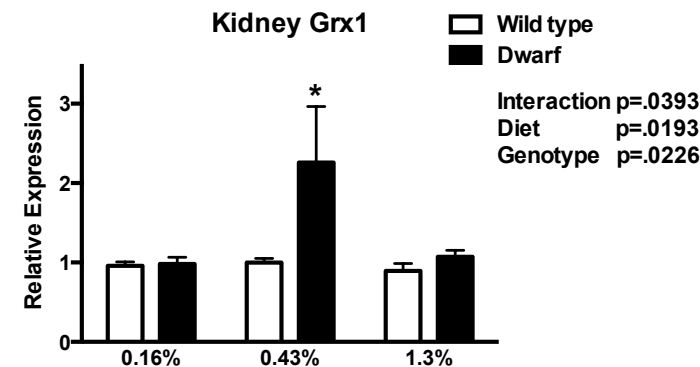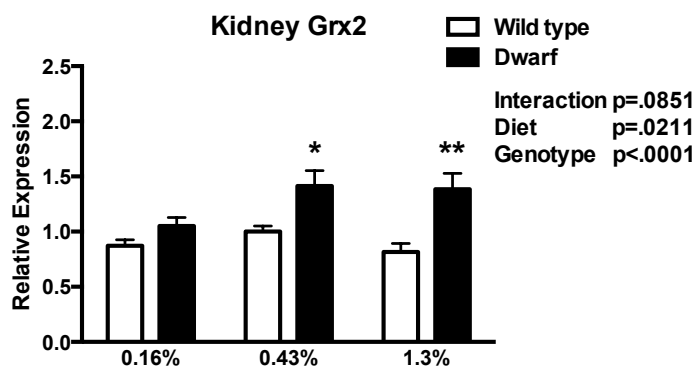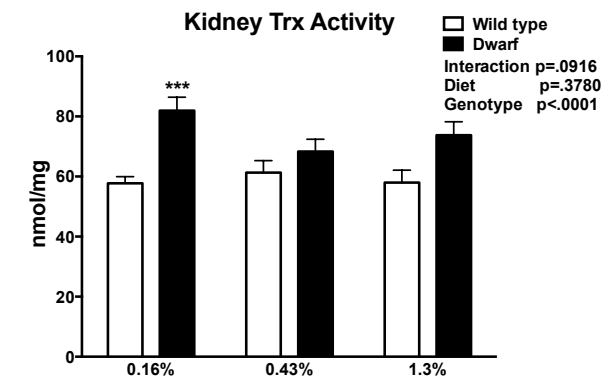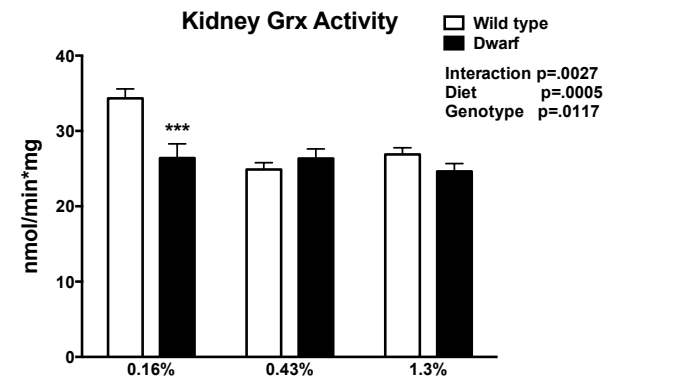

% methionine

% methionine

Supplement: Supplementary file 8 — Additional file 8: Figure S8: Gene expression (relative expression) and activity (nmol/min*mg protein) levels of kidney glutaredoxin (Grx) and thioredoxin (Trx, TrxR) in Ames dwarf and wild-type mice following 8 weeks of dietary methionine consumption. Values represent means ± SEM (n = 10–16/genotype/diet). *p < 0.05, **p < 0.01, ***p < 0.001, and ****p < 0.0001 dwarf versus wild-type mice. (PDF 67 KB) [file 13685_2014_31_MOESM8_ESM.pdf]

Muscle Trx1

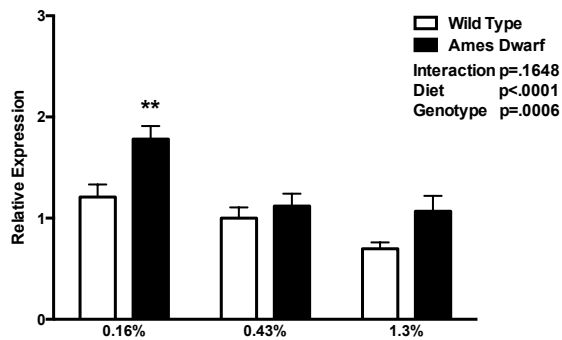

Muscle Trx2

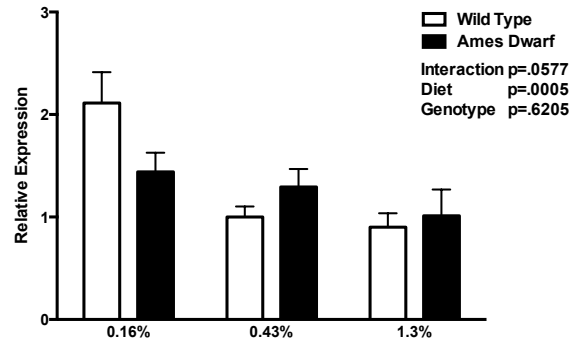

Muscle TrxR1

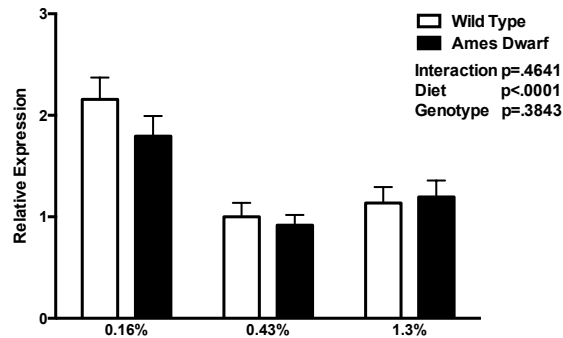

Muscle TrxR2

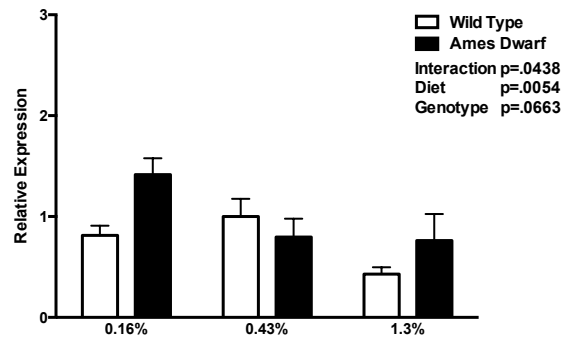

Muscle Grx1

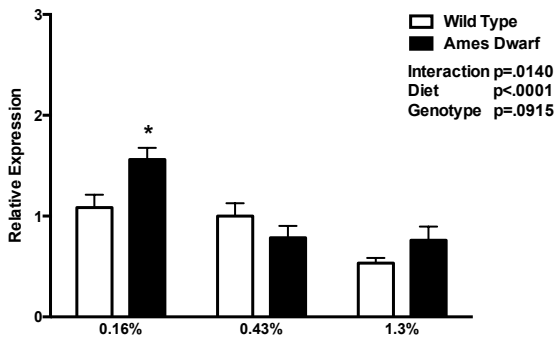

% methionine

Muscle Grx2

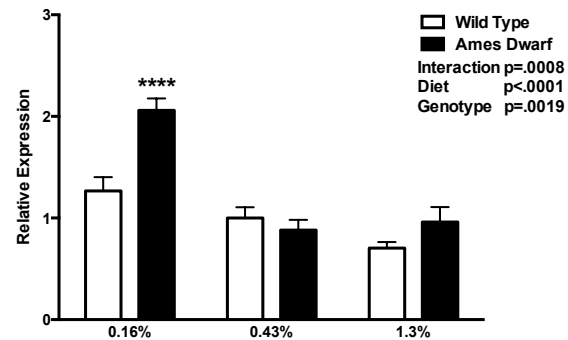

% methionine

Supplement: Supplementary file 10 — Additional file 10: Figure S10: Gene expression (relative expression) levels of skeletal muscle glutaredoxin (Grx) and thioredoxin (Trx, TrxR) in Ames dwarf and wild-type mice following 8 weeks of dietary methionine consumption. Values represent means ± SEM (n = 10–16/genotype/diet). *p < 0.05, **p < 0.01, and ****p < 0.0001 dwarf versus wild-type mice. (PDF 30 KB) [file 13685_2014_31_MOESM10_ESM.pdf]

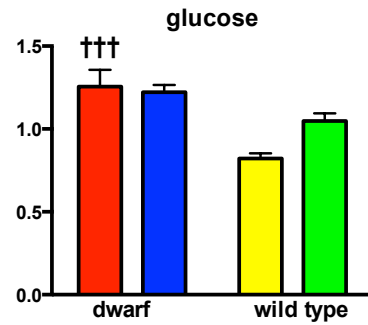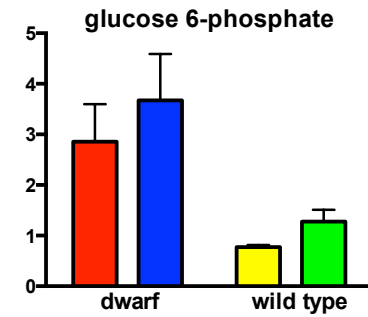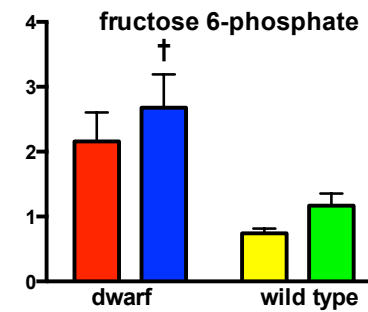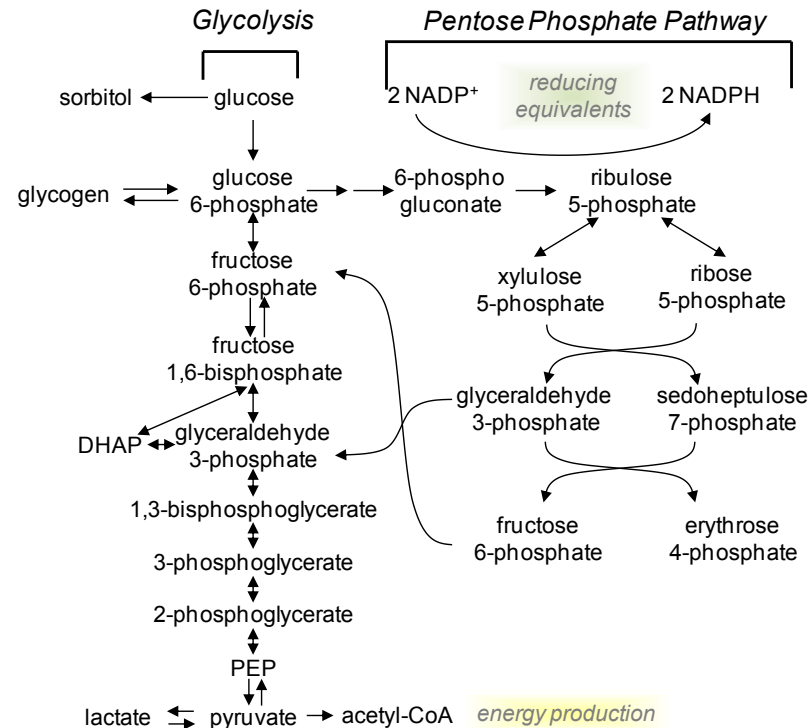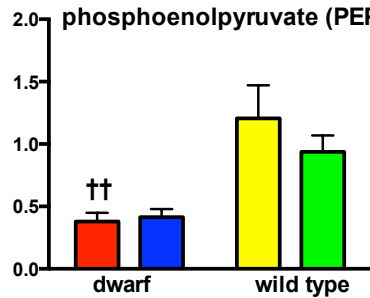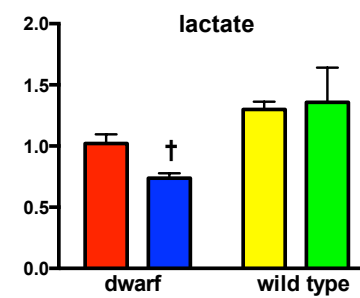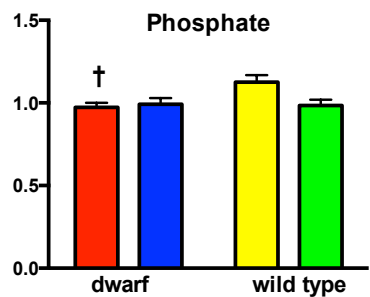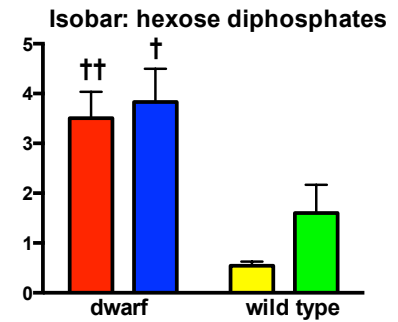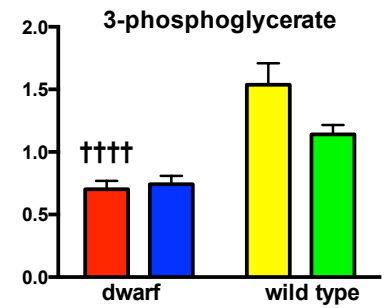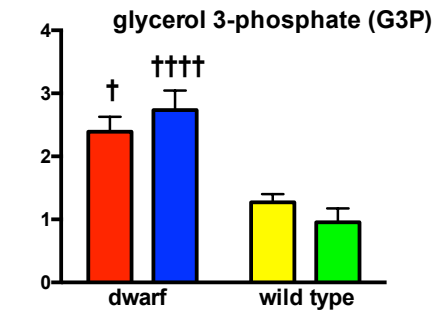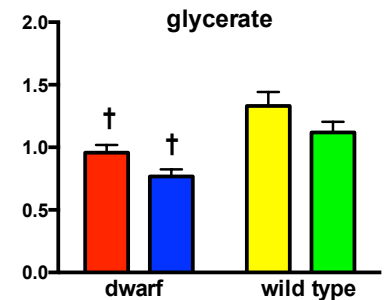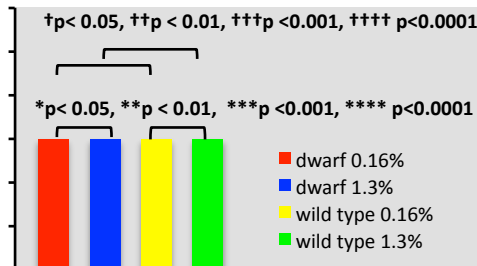

Supplement: Supplementary file 11 — Additional file 11: Figure S11: Liver glycolysis and pentose phosphate metabolites (scaled intensity) in Ames dwarf and wild-type mice following 8 weeks of 0.16% (red/yellow) or 1.3% (blue/green) dietary methionine consumption. Asterisks represent significant differences between diets within a genotype. Crosses represent significant genotype differences within a diet. Values represent means ± SEM (n = 8/genotype/diet). (PDF 208 KB) [file 13685_2014_31_MOESM11_ESM.pdf]

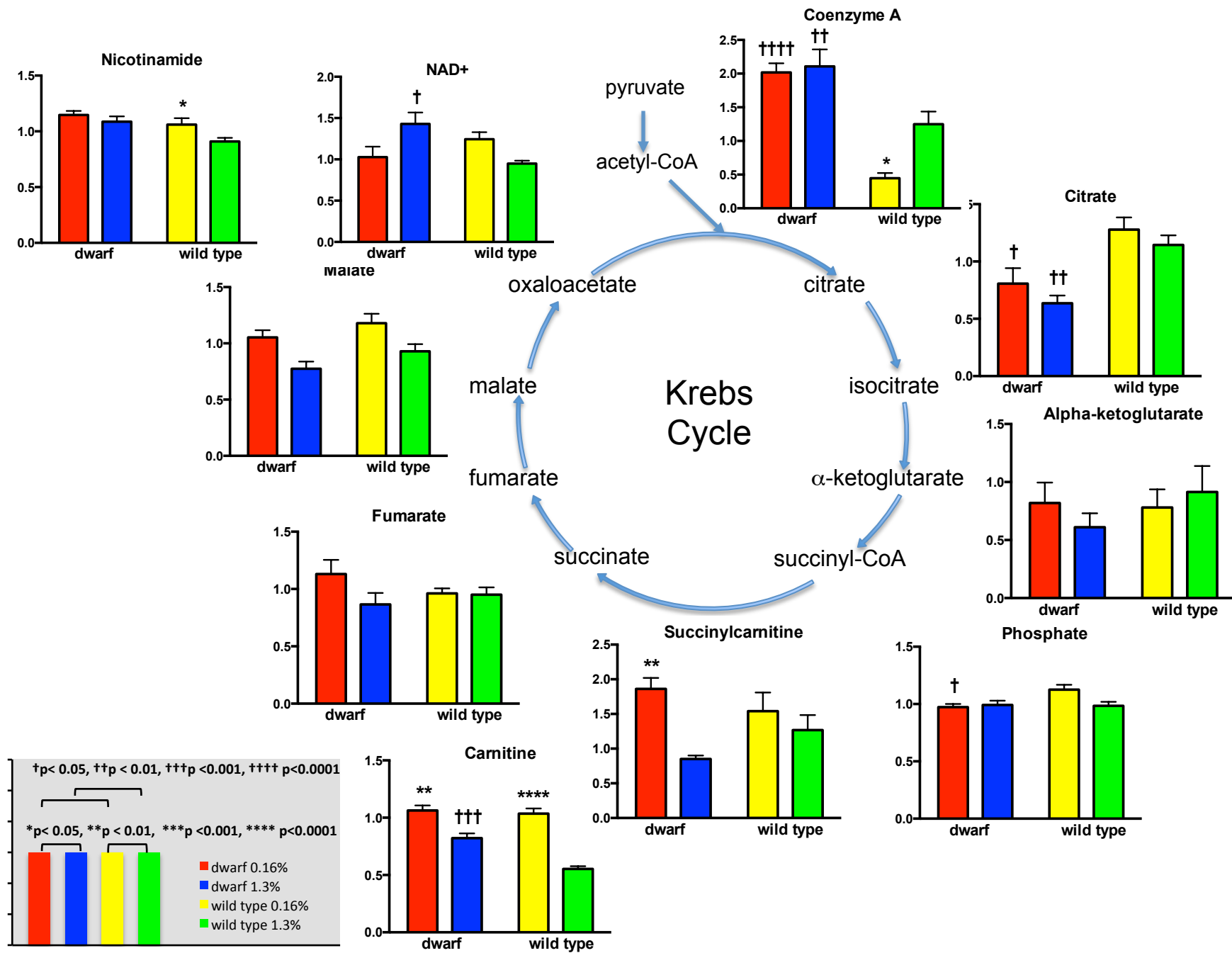

Supplement: Supplementary file 12 — Additional file 12: Figure S12: Liver Krebs cycle metabolites (scaled intensity) in Ames dwarf and wild-type mice following 8 weeks of 0.16% (red/yellow) or 1.3% (blue/green) dietary methionine consumption. Asterisks represent significant differences between diets within a genotype. Crosses represent significant genotype differences within a diet. Values represent means ± SEM (n = 8/genotype/diet). (PDF 214 KB) [file 13685_2014_31_MOESM12_ESM.pdf]
