# Supplementary material for: Altered dietary methionine differentially impacts glutathione and methionine metabolism in long-living growth hormone-deficient Ames dwarf and wild-type mice
Source: Longev Healthspan. 2014 Dec 15;3:10. doi: 10.1186/2046-2395-3-10 (PMC4290132; doi:10.1186/2046-2395-3-10)
Supplement: Supplementary file 4 — Additional file 4: Table S4: Liver, kidney, and skeletal muscle glutathione (GSH) and glutathione disulfide (GSSG) in Ames dwarf and wild-type mice following 8 weeks of dietary methionine consumption (0.16%, 0.43%, 1.3%). Values represent means ± SEM (n = 10–16/genotype/diet). *p < 0.05, **p < 0.01, and ****p < 0.0001 dwarf versus wild-type mice. (PDF 61 KB) [file 13685_2014_31_MOESM4_ESM.pdf]

Additional file 4. Tissue GSH, GSSG and GSH/GSSG ratios from Ames dwarf and wild type mice.

Values represent mean ± SEM (n). P values represent results of a two-way ANOVA.

Genotype tissue	Dwarf 0.16%	Wild type 0.16%	Dwarf 0.43%	Wild type 0.43%	Dwarf 1.3%	Wild type 1.3%	P value Genotype	P value diet	P value interaction
**Liver GSH (n)**	**1499 ± 35 (13)**	**1123 ± 65 (13)**	**1789 ±77 (12)**	**2824 ± 123 (12)**	**1740 ± 84 (11)**	**2677 ± 121 (13)**	**<.0001**	**<.0001**	**<.0001**
**Liver GSSG (n)**	**29.3 ± 1.9 (13)**	**30.5 ± 2.7 (13)**	**21.3 ± 1.0 (12)**	**44.8 ±4.1 (12)**	**28.6 ± 3.5 (11)**	**46.4 ± 4.5 (13)**	**.0001**	**.0207**	**.0307**
**Liver GSH:GSSG**	**54.2 ± 4.3 (13)**	**38.9 ± 2.4 (13)**	**57.5 ± 2.6 (12)**	**69.8 ± 7.6 (12)**	**71.9 ± 10.0 (11)**	**63.8 ± 6.4 (13)**	**.4537**	**.0015**	**.0652**
**Kidney GSH (n)**	**23.07 ± 3.3 (16)**	**15.11 ± 0.5 (16)**	**39.9 ± 3.4 (12)**	**32.08 ± 3.0 (11)**	**29.66 ± 2.8 (11)**	**21.29 ± 1.4 (13)**	**.0003**	**<.0001**	**.9953**
**Kidney GSSG (n)**	**7.15 ± 0.3 (12)**	**6.48 ± 0.3 (16)**	**6.09 ± 0.4 (12)**	**6.18 ± 0.4 (11)**	**7.15 ± 0.6 (10)**	**6.91 ± 0.6 (13)**	**.5138**	**.2061**	**.7409**
**Kidney GSH:GSSG**	**3.54 ± 0.5 (12)**	**2.45 ± 0.2 (16)**	**6.93 ± 0.8 (12)**	**6.31 ± 1.1 (11)**	**4.39 ± 0.4 (10)**	**3.25 ± 0.3 (13)**	**.0702**	**<.0001**	**.9157**
**Skeletal Muscle GSH (n)**	**182.06 ± 9.5 (16)**	**190.68 ± 9.0 (15)**	**180.73 ± 7.3 (12)**	**204.77 ± 6.2 (14)**	**174.98 ± 5.8 (11)**	**186.91 ± 7.7 (13)**	**.0279**	**.3758**	**.6014**
**Skeletal Muscle GSSG (n)**	**21.29 ± 1.0 (16)**	**19.27 ± 1.5 (16)**	**17.75 ± 1.3 (12)**	**18.8 ± 0.8 (12)**	**18.25 ± 0.8 (11)**	**15.87 ± 1.2 (13)**	**.2653**	**.0230**	**.3157**
**Skeletal Muscle GSH:GSSG**	**8.85 ± 0.7 (16)**	**10.64 ± 1.0 (15)**	**10.79 ± 0.9 (12)**	**10.86 ± 0.4 (12)**	**9.76 ± 0.5 (11)**	**12.90 ± 1.3 (13)**	**.0228**	**.1709**	**.2502**
