# Supplementary material for: Altered dietary methionine differentially impacts glutathione and methionine metabolism in long-living growth hormone-deficient Ames dwarf and wild-type mice
Source: Longev Healthspan. 2014 Dec 15;3:10. doi: 10.1186/2046-2395-3-10 (PMC4290132; doi:10.1186/2046-2395-3-10)
Supplement: Supplementary file 5 — Additional file 5: Supporting information. Supporting text for Additional files 6, 7, 8, 9, and 10 describing the kidney and muscle tissue responses to altered MET diet consumption. (PDF 50 KB) [file 13685_2014_31_MOESM5_ESM.pdf]

The kidney plays a lessor role in generating Met but contributes functionally in terms of metabolism, detoxification and stress resistance. Mat was elevated in dwarf compared to wild type mice. Marked differences were observed in genotypes on the 0.16% MET compared to the other diets. Dwarf mouse kidney Gnmt mRNA levels were higher than wild type mice across diets similar to liver. Ahcy was increased in dwarf kidney on all diets in comparison to wild type mice. Both genotypes exhibited lower levels of Ahcy when consuming the 1.3% MET diet. Diet and genotype affected the methionine recycling enzymes. Dwarf mice expressed higher levels of Mtr, Mthfr and Bhmt compared to wild type mice. Diet was a significant factor in the expression of Cbs and Cth. Dwarf mice expressed higher levels of the Cbs and Cth proteins in the 0.16% MET group when compared to wild type mice (data not shown). Biosynthesis of GSH occurs primarily in liver tissue but levels of the Gclm subunit mRNA were lower in dwarf kidney in MET restriction compared to wild type mice. GSH levels were increased in dwarf mice and diet appeared to impact GSH in this tissue. The GSH:GSSG ratios were influenced by diet. The majority of GGT activity occurs in kidney and the dwarf exhibited less GGT activity compared to wild type mice. Dwarf mice exhibited higher GST activities compared to wild type mice regardless of diet. Expression of Trx1, Trx2, TrxR1 and TrxR2 were higher in dwarf kidneys across diets and most increased with increasing MET levels. Trx activity was affected by genotype, reflecting the high levels of mRNA expression. Grx1 and Grx2 also tended to be greater in dwarf mice when compared to wild types however the activity of Grx was lower or similar to that of the wild type mice.

The tissue distribution of transsulfuration is limited and particularly active in liver however, kidney tissue also contains the necessary enzymes but at lower levels, plus kidney plays a key role in recycling of cysteine via degradation of GSH by GGT [supporting references 1-3]. Our

previous work showed highly elevated kidney GGT activities in dwarf compared to wild type mice [44]. Under the conditions of the current study, however overall GGT activity was lower in dwarf mice on each of the diets. The MET pathway expression in the kidney was similar to that observed in hepatic tissue with higher levels of each of these enzymes in dwarf versus wild type mice regardless of diet (one exception-Mtr 0.16%). These differences are reflected in higher kidney GSH levels, GST activity and redoxin levels in dwarf mice regardless of dietary MET level. In general, the trends observed in hepatic MET and GSH pathways were maintained in kidney tissues.

Muscle tissue was evaluated as a control tissue in which MET metabolic activity is low. Skeletal muscle tissue Mat1a expression was high in dwarf mice on MET restriction. Gnmt mRNA levels were increased in dwarf mice and decreased with increasing MET content. Ahcy, Mtr, Bhmt and Mthfr mRNA levels were all higher in the dwarf on MET restriction compared to wild type mice. Differences between genotypes were not as apparent on higher levels of MET. Transsulfuration genes in muscle tissue were impacted by genotype and diet. Small but significant decreases in Gclc and Gclm were observed between 0.16% and 1.3% diets whereas genotype was not a factor. Skeletal muscle GSH was lower in dwarf mice but not affected by diet. Much like liver and kidney, redoxins were elevated in skeletal muscles in dwarf mice on the 0.16% MET diets. Similar to other genes, the levels of each declined in both genotypes with increasing MET content. In general, genotype differences were reflected across tissues.

Supporting references

1. Stipanuk MH, De la Rosa J, Hirschberger LL: **Catabolism of cyst(e)ine by rat renal cortical tubules.** *J Nutr* 1990, **120**:450-458.
2. Finkelstein JD: **Methionine metabolism in mammals.** *J Nutr Biochem* 1990, **1**:228-237.

3. Slow S, Garrow TA: **Liver choline dehydrogenase and kidney betaine-homocysteine methyltransferase expression are not affected by methionine or choline intake in taking rats.** *J Nutr* 2006, **136**:2279-2286.
