# Supplementary material for: Altered dietary methionine differentially impacts glutathione and methionine metabolism in long-living growth hormone-deficient Ames dwarf and wild-type mice
Source: Longev Healthspan. 2014 Dec 15;3:10. doi: 10.1186/2046-2395-3-10 (PMC4290132; doi:10.1186/2046-2395-3-10)
Supplement: Supplementary file 9 — Additional file 9: Figure S9: Skeletal muscle gene expression (relative expression) of methionine pathway components in Ames dwarf and wild-type mice following 8 weeks of 0.16%, 0.43%, or 1.3% dietary methionine consumption. Values represent means ± SEM (n = 10–16/genotype/diet). *p < 0.05, **p < 0.01, ***p < 0.001, and ****p < 0.0001 dwarf versus wild-type mice. (PDF 38 KB) [file 13685_2014_31_MOESM9_ESM.pdf]

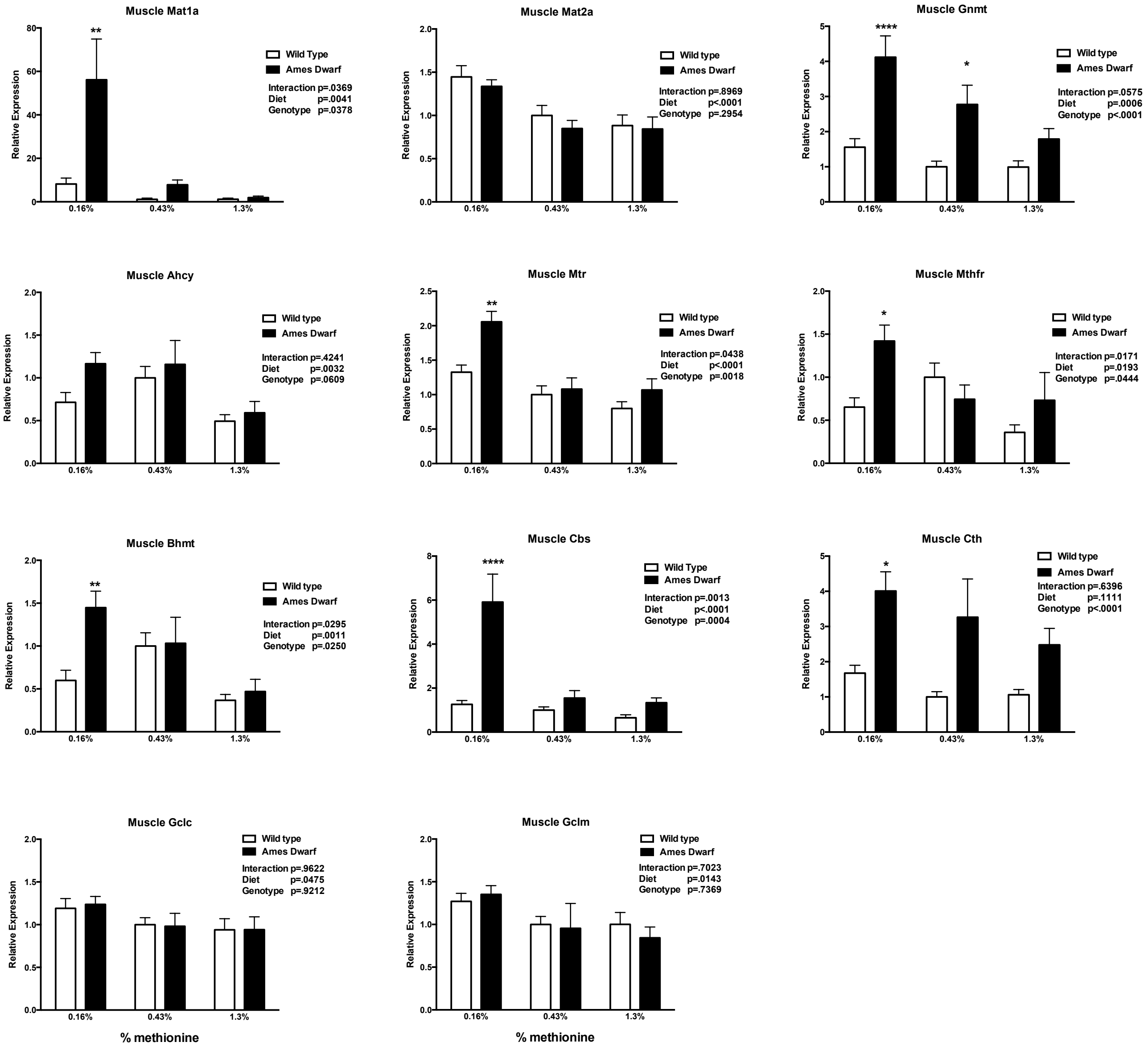

Muscle Mat1a
Wild Type
Ames Dwarf
Interaction p=.0369
Diet p=.0041
Genotype p=.0378
Muscle Mat2a
Wild type
Ames Dwarf
Interaction p=.8969
Diet p<.0001
Genotype p=.2954
Muscle Gnmt
Wild type
Ames Dwarf
Interaction p=.0575
Diet p=.0006
Genotype p<.0001
Muscle Ahcy
Wild type
Ames Dwarf
Interaction p=.4241
Diet p=.0032
Genotype p=.0609
Muscle Mtr
Wild type
Ames Dwarf
Interaction p=.0438
Diet p<.0001
Genotype p=.0018
Muscle Mthfr
Wild type
Ames Dwarf
Interaction p=.0171
Diet p=.0193
Genotype p=.0444
Muscle Bhmt
Wild type
Ames Dwarf
Interaction p=.0295
Diet p=.0011
Genotype p=.0250
Muscle Cbs
Wild Type
Ames Dwarf
Interaction p=.0013
Diet p<.0001
Genotype p=.0004
Muscle Cth
Wild type
Ames Dwarf
Interaction p=.6396
Diet p=.1111
Genotype p<.0001
Muscle Gclc
Wild type
Ames Dwarf
Interaction p=.9622
Diet p=.0475
Genotype p=.9212
Muscle Gclm
Wild type
Ames Dwarf
Interaction p=.7023
Diet p=.0143
Genotype p=.7369
Relative Expression
0.16%
0.43%
1.3%
% methionine
